# Supplementary figures and images for: Plasma membrane remodeling in GM2 gangliosidoses drives synaptic dysfunction
Source: PLoS Biol. 2025 Jul 3;23(7):e3003265. doi: 10.1371/journal.pbio.3003265 (PMC12251256; doi:10.1371/journal.pbio.3003265)

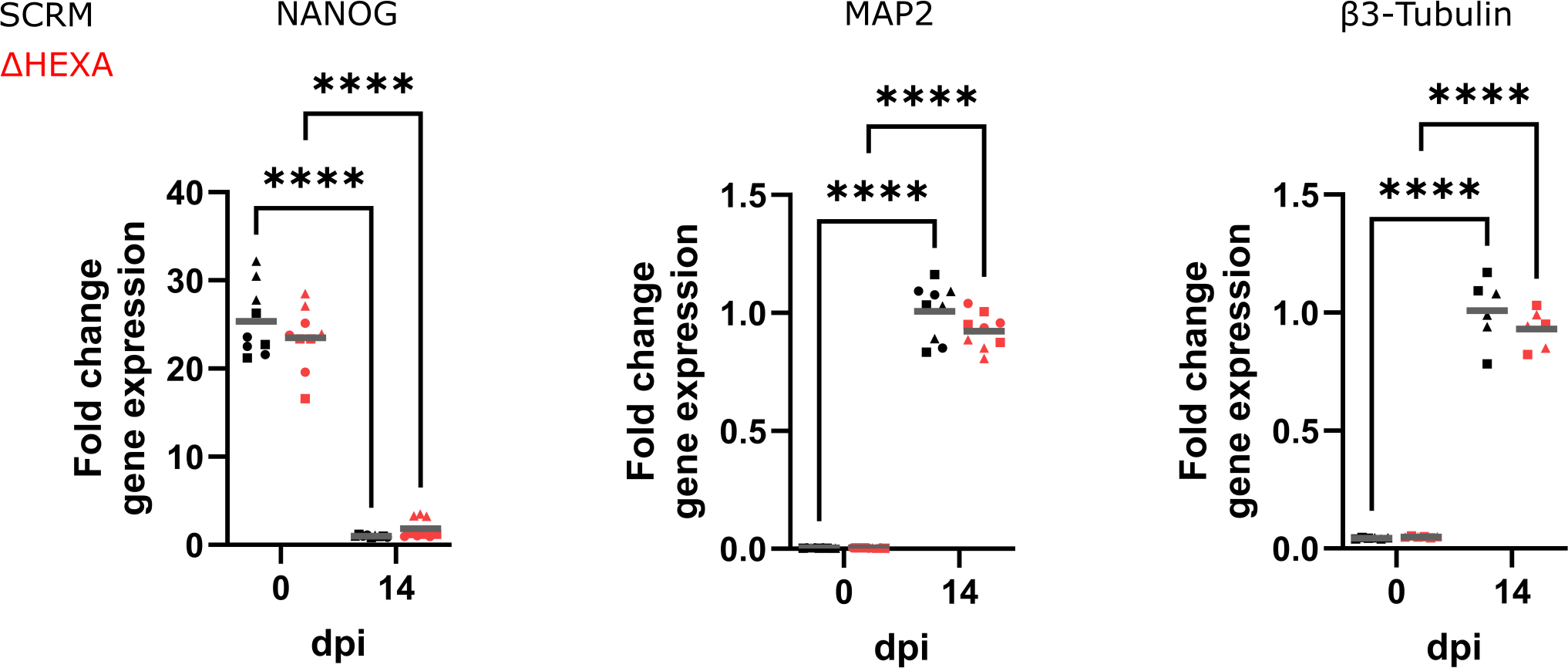

Supplement: S1 Fig — Fold change is calculated relative to 14 dpi SCRM controls, N = 3 biological replicates for NANOG and MAP2 and N = 2 biological replicates for β3-Tubulin were carried out in technical triplicate n = 3 (squares, triangles, circles) and the mean is displayed (grey lines). Significance was determined with a two-way ANOVA, ****p ≤ 0.0001. Underlying data used to generate these figures are available in S1 Data at https://doi.org/10.17863/CAM.118836. (TIF) [file pbio.3003265.s001.tif]

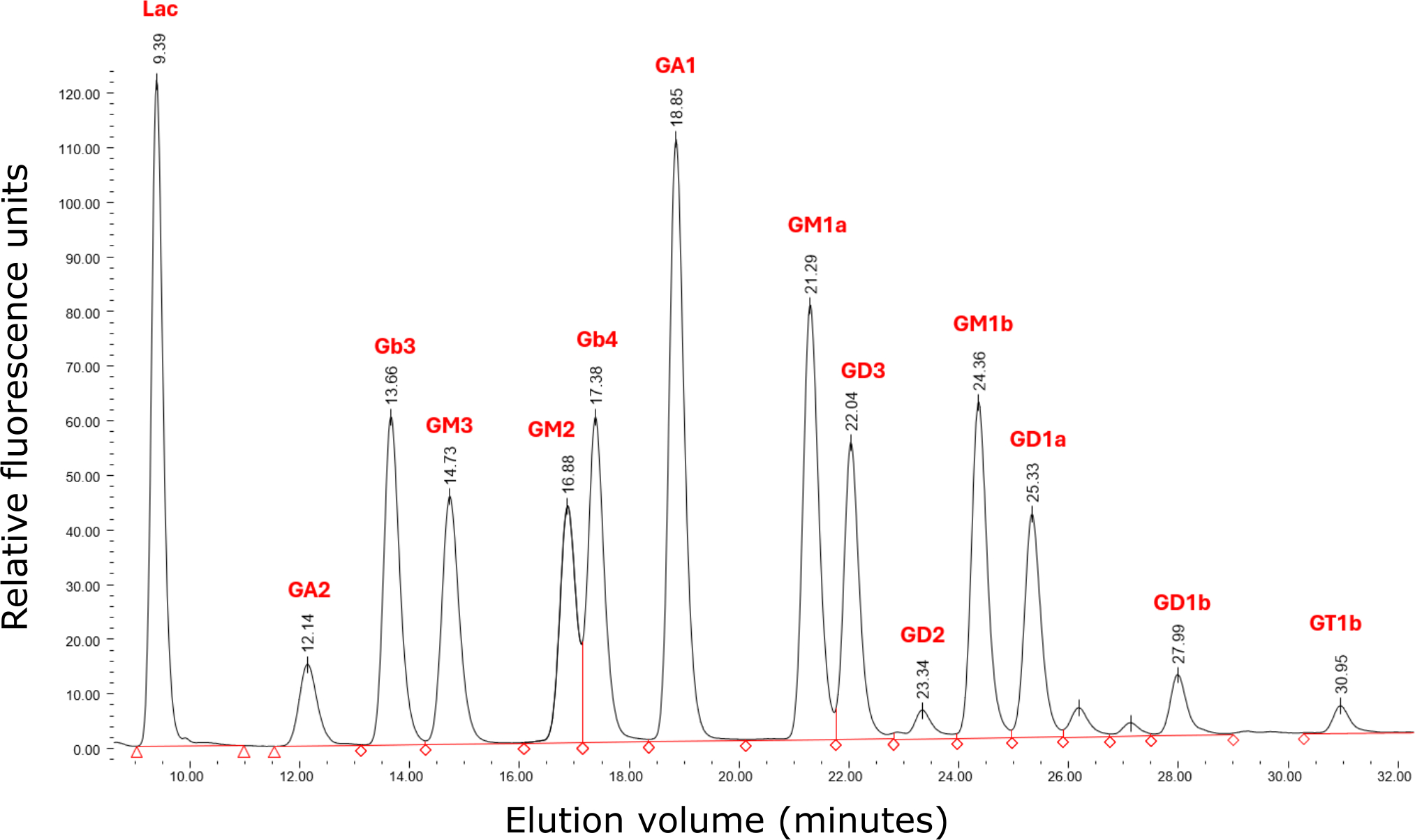

Supplement: S2 Fig — Elution profile of known GSL standards as reference for peak identification. Each glycan headgroup has a distinct elution volume (labelled). (TIF) [file pbio.3003265.s002.tif]

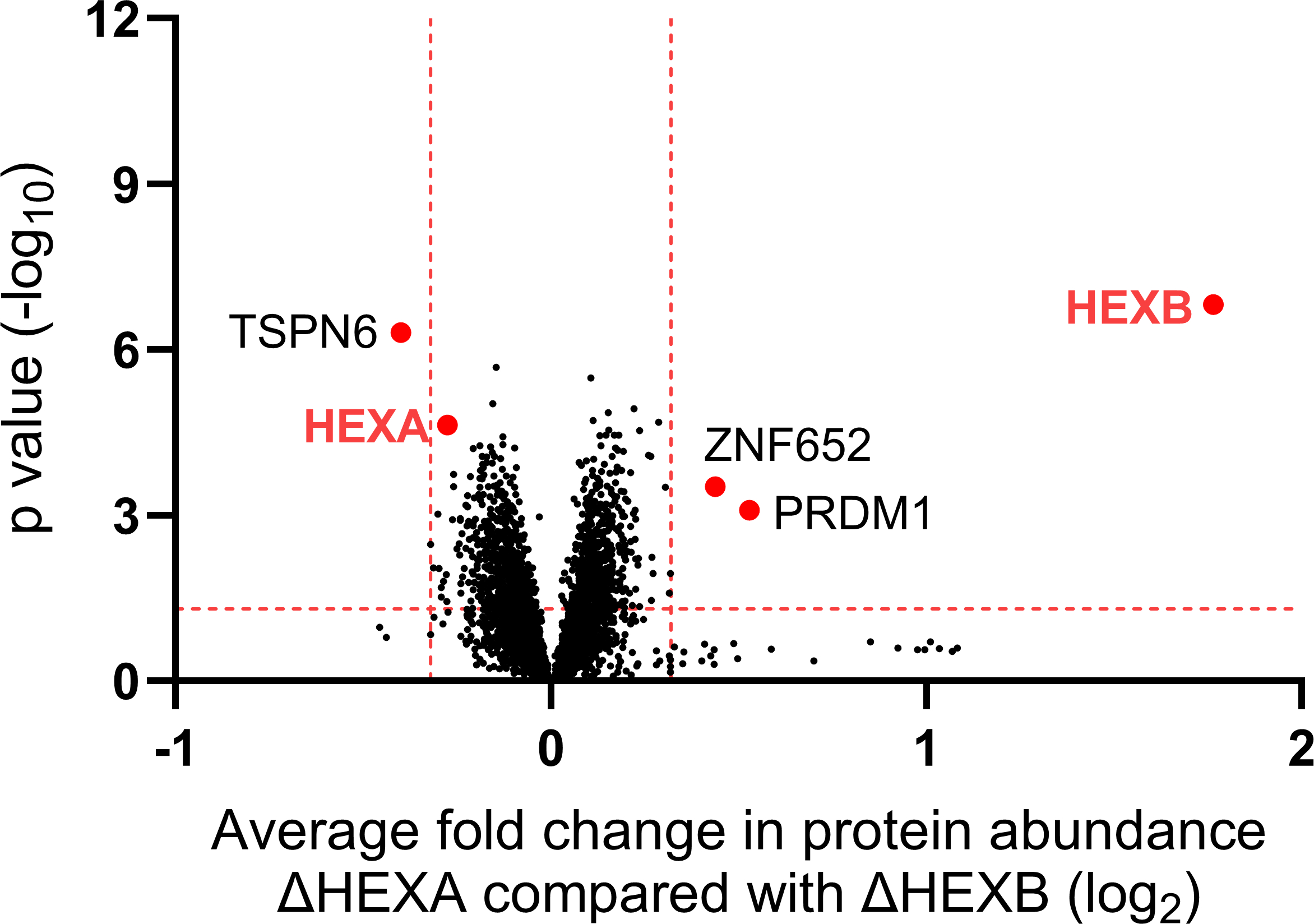

Supplement: S3 Fig — A volcano plot is shown with the horizontal axis showing average fold change across three biological replicates and the vertical axis showing significance (two-sided t test) across the three replicates. Significance cutoffs of >20% change and p-value < 0.05 are indicated (red dotted lines). Beyond the subunits that have been knocked down there are only 3 proteins with significant changes in protein abundance between these cell lines. Underlying data used to generate these figures are available in S1 Data at https://doi.org/10.17863/CAM.118836. (TIF) [file pbio.3003265.s003.tif]

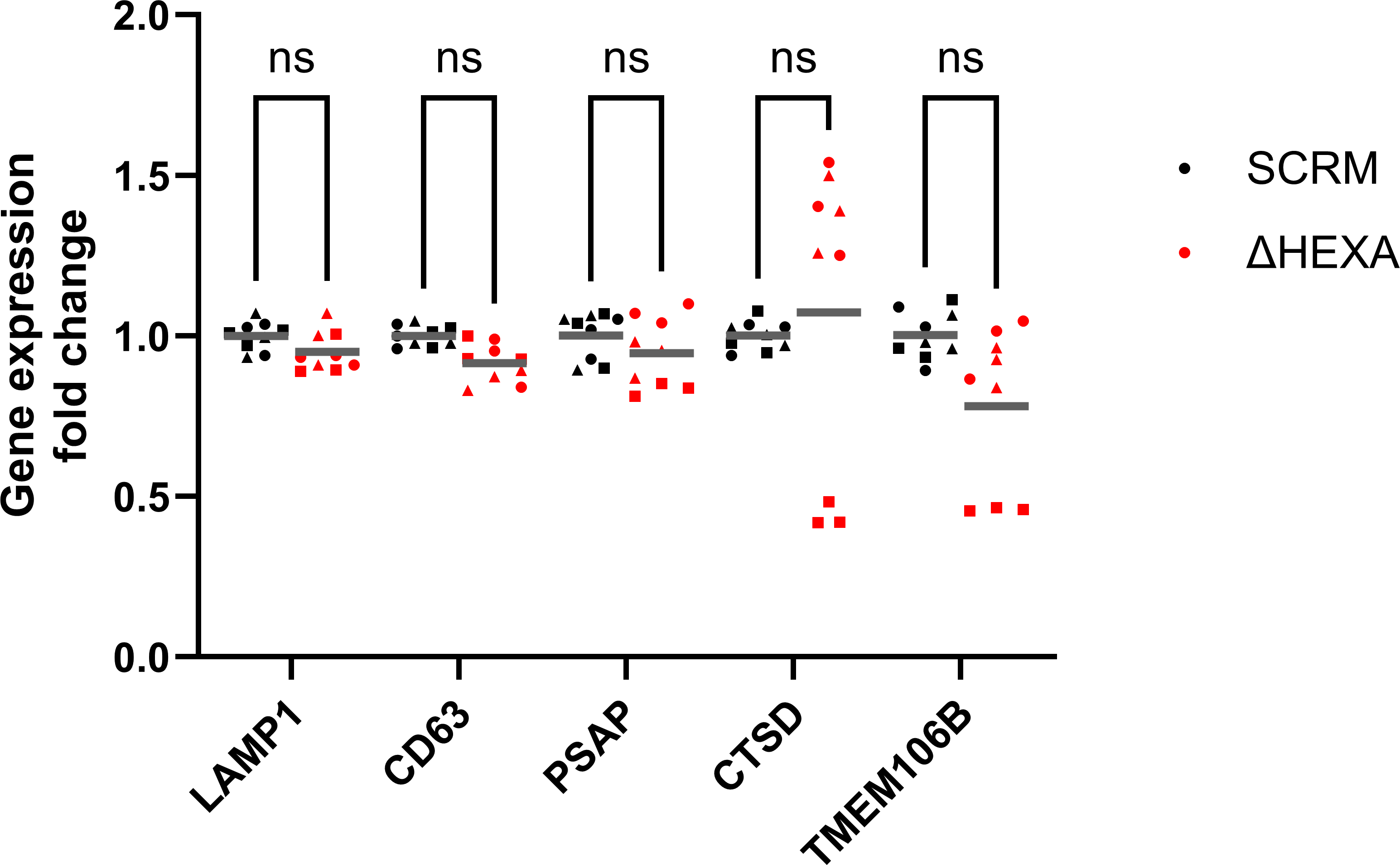

Supplement: S4 Fig — Gene expression levels are shown at 14 dpi for SCRM and ΔHEXA lines. Fold change is calculated relative to SCRM controls, N = 3 biological replicates were carried out in technical triplicate n = 3 (squares, triangles, circles) and the mean is displayed (grey line). Significance was determined with a one-way ANOVA, no significant (ns) differences are seen between SCRM and ΔHEXA lines. Underlying data used to generate these figures are available in S1 Data at https://doi.org/10.17863/CAM.118836. (TIF) [file pbio.3003265.s004.tif]

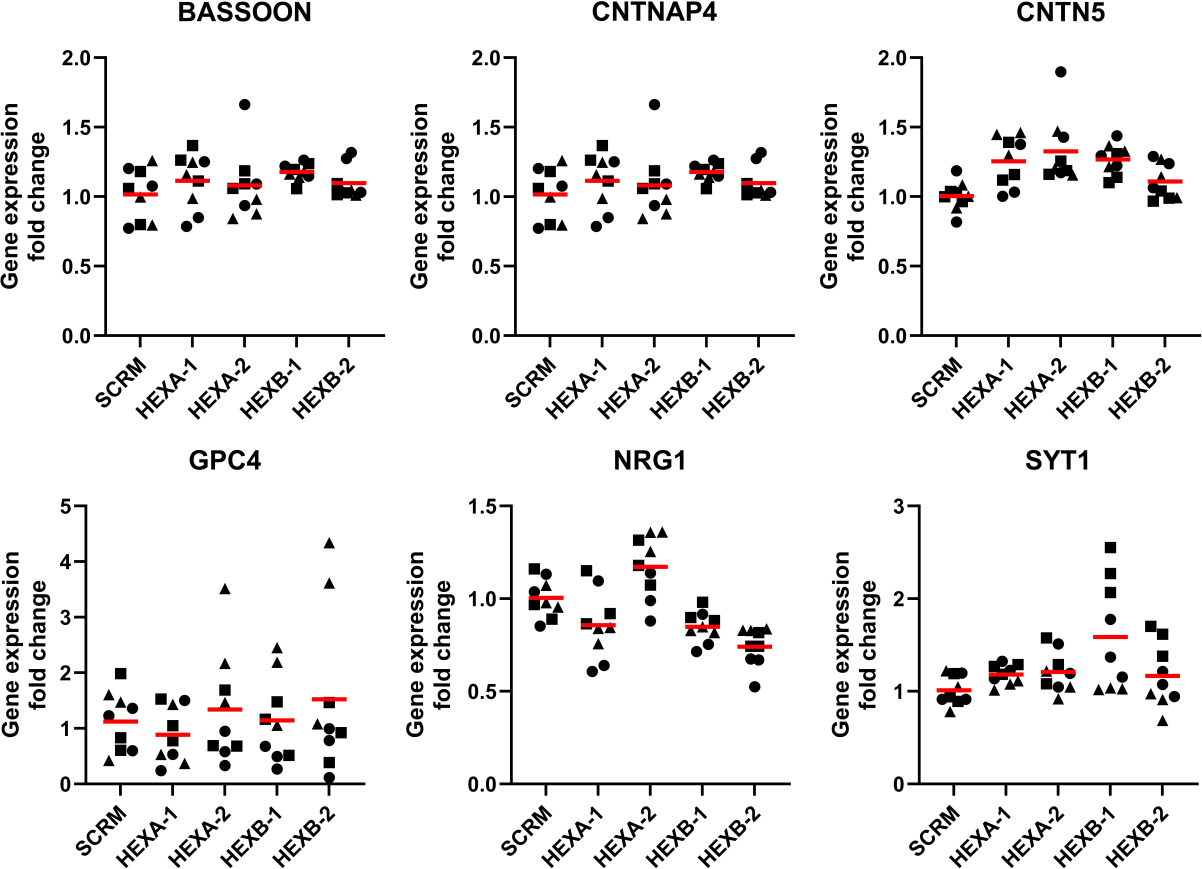

Supplement: S5 Fig — Fold change is calculated relative to SCRM controls, N = 3 biological replicates were carried out in technical triplicate n = 3 (squares, triangles, circles) and the mean is displayed (red line). Significance was carried out using a one-way ANOVA. Although some changes were determined to be significant in these data, the fold change was less than 2-fold meaning it did not satisfy the criteria for a reliable fold change by qPCR [137]. Underlying data used to generate these figures are available in S1 Data at https://doi.org/10.17863/CAM.118836. (TIF) [file pbio.3003265.s005.tif]

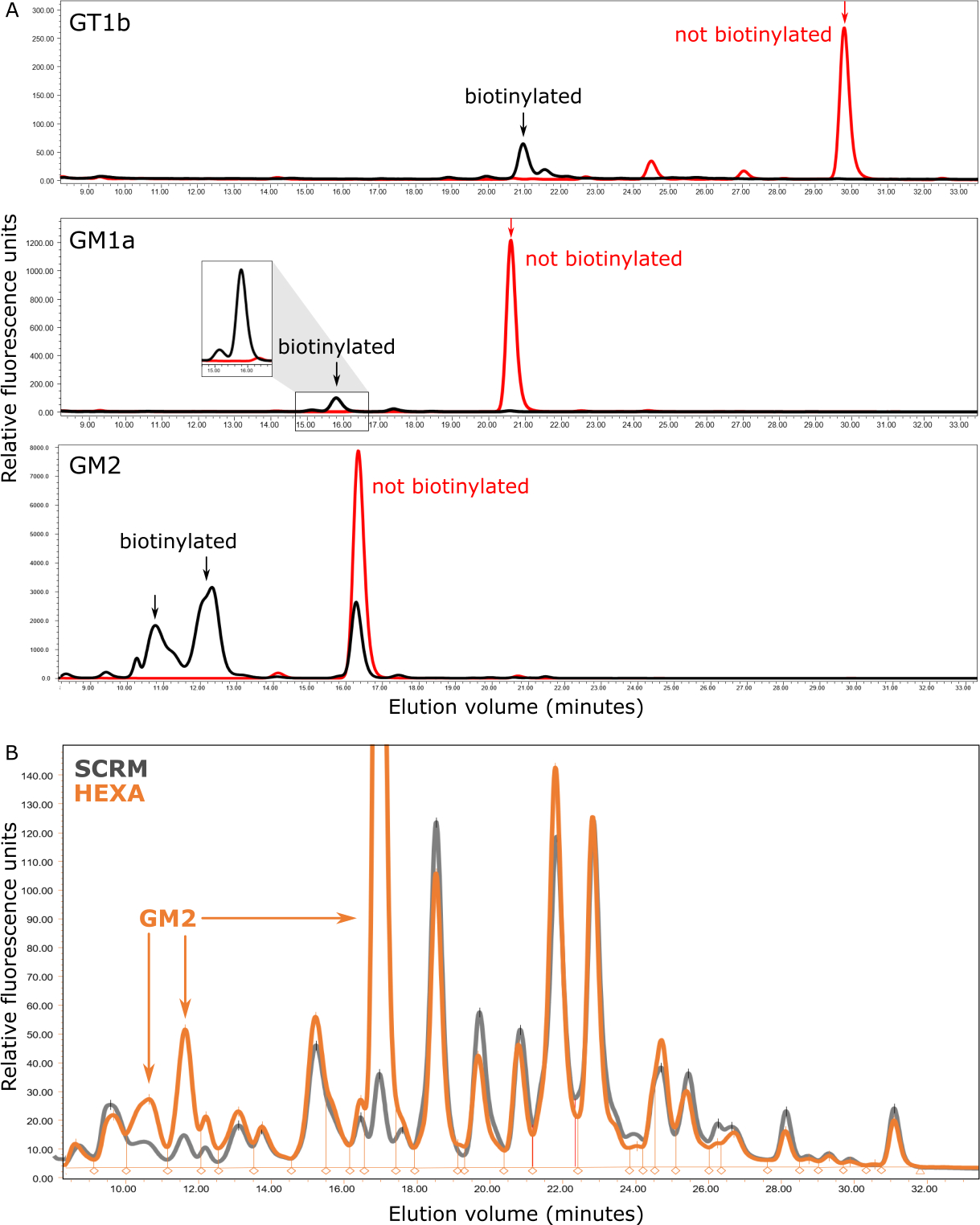

Supplement: S6 Fig — A. Standards from individually labelled lipid species GT1b, GM1a and GM2. B. Ganglioside headgroup elution profiles of 28 dpi whole cell samples after surface labelling with aminoxybiotin. Underlying data used to generate these figures are available in S1 Data at https://doi.org/10.17863/CAM.118836. (TIF) [file pbio.3003265.s006.tif]
