# Supplementary material for: Plasma membrane remodeling in GM2 gangliosidoses drives synaptic dysfunction
Source: PLoS Biol. 2025 Jul 3;23(7):e3003265. doi: 10.1371/journal.pbio.3003265 (PMC12251256; doi:10.1371/journal.pbio.3003265)
Supplement: S1 Table — (DOCX) [file pbio.3003265.s007.docx]

**S1 Table.** Guide RNA target sequences

| Gene | Exon | Target sequence |
| --- | --- | --- |
| HEXA-1 | 1 | 5’ - CAGGTCACGATAGCGCTGGA |
| HEXA-2 | 1 | 5’ - CCAAAGCCTGGAGCTTGTCA |
| HEXB-1 | 1 | 5’ - GCTGTTGGCGACACTGCTGG |
| HEXB-2 | Prior to 1 | 5’ - CCGCTCGGCTGCTTTCCGCC |
| GLB-1 | 1 | 5’ - ACAGAGGGAGGATGCGAACC |
| SCRM | N/A | 5’ - GGGACGCGAAAGAAACCAGT |
