# Supplementary material for: Plasma membrane remodeling in GM2 gangliosidoses drives synaptic dysfunction
Source: PLoS Biol. 2025 Jul 3;23(7):e3003265. doi: 10.1371/journal.pbio.3003265 (PMC12251256; doi:10.1371/journal.pbio.3003265)
Supplement: S2 Table — (DOCX) [file pbio.3003265.s008.docx]

**S2 Table.** High confidence targets identified in WCP of ΔHEXA and ΔHEXB compared with SCRM control cells at 14 dpi.

| **Gene ID** | **Description** | **Log_2_ Fold change** | **Significance p-value** |
| --- | --- | --- | --- |
| O60637 | Tetraspanin-3 GN=TSPAN3 | 1.532 | 1.2E-09 |
| Q9C0H2 | Protein tweety homolog 3 GN=TTYH3 | 1.088 | 2.9E-09 |
| P38571 | Lysosomal acid lipase/cholesteryl ester hydrolase GN=LIPA | 0.980 | 3.0E-05 |
| O43657 | Tetraspanin-6 GN=TSPAN6 | 0.968 | 1.0E-05 |
| Q14108 | Lysosome membrane protein 2 GN=SCARB2 | 0.879 | 1.7E-09 |
| Q7Z3F1 | Integral membrane protein GPR155 GN=GPR155 | 0.778 | 5.1E-05 |
| P13645 | Keratin, type I cytoskeletal 10 GN=KRT10 | 0.763 | 3.5E-02 |
| Q9BXS4 | Transmembrane protein 59 GN=TMEM59 | 0.746 | 2.8E-08 |
| Q9BT67 | NEDD4 family-interacting protein 1 GN=NDFIP1 | 0.738 | 3.3E-05 |
| P08962 | CD63 antigen GN=CD63 | 0.730 | 9.6E-09 |
| P50897 | Palmitoyl-protein thioesterase 1 GN=PPT1 | 0.663 | 2.7E-05 |
| Q9H6Y7 | E3 ubiquitin-protein ligase RNF167 GN=RNF167 | 0.662 | 1.3E-08 |
| P31944 | Caspase-14 GN=CASP14 | 0.657 | 4.7E-02 |
| P61916 | Epididymal secretory protein E1 GN=NPC2 | 0.604 | 5.5E-06 |
| Q99758 | ATP-binding cassette sub-family A member 3 GN=ABCA3 | 0.563 | 2.5E-08 |
| O43567 | E3 ubiquitin-protein ligase RNF13 GN=RNF13 | 0.551 | 9.9E-06 |
| P78382 | CMP-sialic acid transporter GN=SLC35A1 | 0.543 | 8.3E-07 |
| P11279 | Lysosome-associated membrane glycoprotein 1 GN=LAMP1 | 0.488 | 5.8E-09 |
| Q96P63 | Serpin B12 GN=SERPINB12 | 0.481 | 4.9E-02 |
| P41732 | Tetraspanin-7 GN=TSPAN7 | 0.463 | 5.4E-08 |
| Q9NUN5 | Probable lysosomal cobalamin transporter GN=LMBRD1 | 0.462 | 1.9E-11 |
| Q8IY95 | Transmembrane protein 192 GN=TMEM192 | 0.425 | 1.2E-04 |
| P07339 | Cathepsin D GN=CTSD | 0.408 | 3.1E-06 |
| Q9NUM4 | Transmembrane protein 106B GN=TMEM106B | 0.386 | 1.6E-06 |
| Q8NCC5 | Sugar phosphate exchanger 3 GN=SLC37A3 | 0.377 | 1.2E-05 |
| O15118 | Niemann-Pick C1 protein GN=NPC1 | 0.371 | 8.4E-06 |
| P13473 | Lysosome-associated membrane glycoprotein 2 GN=LAMP2 | 0.342 | 2.3E-04 |
| P0CG05 | Ig lambda-2 chain C regions GN=IGLC2 | 0.337 | 2.9E-02 |
| Q9UJX6 | Anaphase-promoting complex subunit 2 GN=ANAPC2 | 0.323 | 5.5E-06 |
| Q96QD8 | Sodium-coupled neutral amino acid transporter 2 GN=SLC38A2 | 0.308 | 3.2E-03 |
| Q13286 | Battenin GN=CLN3 | 0.307 | 3.0E-02 |
| Q12999 | Tetraspanin-31 GN=TSPAN31 | 0.307 | 1.0E-05 |
| Q9UQM7 | Calcium/calmodulin-dependent protein kinase type II subunit alpha GN=CAMK2A | 0.298 | 2.7E-03 |
| O95772 | MLN64 N-terminal domain homolog GN=STARD3NL | 0.283 | 3.5E-04 |
| Q86WC4 | Osteopetrosis-associated transmembrane protein 1 GN=OSTM1 | 0.283 | 2.8E-04 |
| Q8WTV0 | Scavenger receptor class B member 1 GN=SCARB1 | 0.274 | 2.6E-03 |
| P61513 | 60S ribosomal protein L37a GN=RPL37A | -0.281 | 1.0E-03 |
| P04921 | Glycophorin-C GN=GYPC | -0.286 | 9.8E-03 |
| O75943 | Cell cycle checkpoint protein RAD17 GN=RAD17 | -0.297 | 1.0E-02 |
| Q15125 | 3-beta-hydroxysteroid-Delta(8),Delta(7)-isomerase GN=EBP | -0.299 | 3.5E-02 |
| F8WCM5 | Insulin, isoform 2 GN=INS-IGF2 | -0.302 | 1.8E-02 |
| Q96A83 | Collagen alpha-1(XXVI) chain GN=COL26A1 | -0.308 | 2.7E-03 |
| O15344 | E3 ubiquitin-protein ligase Midline-1 GN=MID1 | -0.313 | 9.9E-04 |
| Q9P2G3 | Kelch-like protein 14 GN=KLHL14 | -0.314 | 2.5E-02 |
| Q8WTS6 | Histone-lysine N-methyltransferase SETD7 GN=SETD7 | -0.321 | 2.0E-02 |
| Q96Q91 | Anion exchange protein 4 GN=SLC4A9 | -0.351 | 3.3E-03 |
| Q9Y2D9 | Zinc finger protein 652 GN=ZNF652 | -0.356 | 4.7E-02 |
| P29373 | Cellular retinoic acid-binding protein 2 GN=CRABP2 | -0.382 | 6.7E-04 |
| Q9NYJ7 | Delta-like protein 3 GN=DLL3 | -0.390 | 8.7E-03 |
| Q14831 | Metabotropic glutamate receptor 7 GN=GRM7 | -0.395 | 7.4E-03 |
| O60663 | LIM homeobox transcription factor 1-beta GN=LMX1B | -0.434 | 6.3E-06 |
| P41145 | Kappa-type opioid receptor GN=OPRK1 | -0.472 | 8.2E-04 |
| O14522 | Receptor-type tyrosine-protein phosphatase T GN=PTPRT | -0.498 | 2.6E-04 |
| Q13237 | cGMP-dependent protein kinase 2 GN=PRKG2 | -0.625 | 2.5E-06 |
| P06865 | Beta-hexosaminidase subunit alpha GN=HEXA | -1.045 | 5.2E-08 |
