# Supplementary material for: Plasma membrane remodeling in GM2 gangliosidoses drives synaptic dysfunction
Source: PLoS Biol. 2025 Jul 3;23(7):e3003265. doi: 10.1371/journal.pbio.3003265 (PMC12251256; doi:10.1371/journal.pbio.3003265)
Supplement: S3 Table — (DOCX) [file pbio.3003265.s009.docx]

**S3 Table.** High confidence targets identified in PMP-MS of ΔHEXA and ΔHEXB compared with SCRM control cells at 14dpi.

| **Gene ID** | **Description** | **Log_2_ Fold change** | **Significance p-value** |
| --- | --- | --- | --- |
| Q9C0A0 | Contactin-associated protein-like 4 GN=CNTNAP4 | 1.084 | 3.0E-07 |
| P21583 | Kit ligand GN=KITLG | 1.032 | 6.0E-07 |
| O94779 | Contactin-5 GN=CNTN5 | 0.913 | 1.6E-06 |
| Q14982 | Opioid-binding protein/cell adhesion molecule GN=OPCML | 0.729 | 7.2E-04 |
| Q14108 | Lysosome membrane protein 2 GN=SCARB2 | 0.683 | 4.1E-04 |
| P16671 | Platelet glycoprotein 4 GN=CD36 | 0.649 | 3.3E-02 |
| O43194 | G-protein coupled receptor 39 GN=GPR39 | 0.612 | 4.6E-03 |
| P55283 | Cadherin-4 GN=CDH4 | 0.609 | 3.7E-07 |
| Q86SJ2 | Amphoterin-induced protein 2 GN=AMIGO2 | 0.606 | 2.7E-06 |
| Q16620 | BDNF/NT-3 growth factors receptor GN=NTRK2 | 0.585 | 8.0E-03 |
| O60320 | Protein FAM189A1 GN=FAM189A1 | 0.555 | 1.5E-03 |
| P78333 | Glypican-5 GN=GPC5 | 0.551 | 1.5E-03 |
| P21579 | Synaptotagmin-1 GN=SYT1 | 0.549 | 6.1E-06 |
| Q96ID5 | Immunoglobulin superfamily member 21 GN=IGSF21 | 0.524 | 9.3E-04 |
| Q9H461 | Frizzled-8 GN=FZD8 | 0.513 | 4.7E-03 |
| Q9H3S1 | Semaphorin-4A GN=SEMA4A | 0.508 | 4.1E-04 |
| P78382 | CMP-sialic acid transporter GN=SLC35A1 | 0.504 | 7.5E-05 |
| O00451 | GDNF family receptor alpha-2 GN=GFRA2 | 0.475 | 8.7E-03 |
| Q13286 | Battenin GN=CLN3 | 0.460 | 2.0E-02 |
| Q9UHI5 | Large neutral amino acids transporter SS 2 GN=SLC7A8 | 0.452 | 9.2E-04 |
| P38571 | Lysosomal acid lipase/cholesteryl ester hydrolase GN=LIPA | 0.446 | 6.9E-03 |
| P39086 | Glutamate receptor ionotropic, kainate 1 GN=GRIK1 | 0.443 | 4.4E-04 |
| O94933 | SLIT and NTRK-like protein 3 GN=SLITRK3 | 0.430 | 1.2E-03 |
| O60637 | Tetraspanin-3 GN=TSPAN3 | 0.426 | 2.8E-03 |
| Q8WXS5 | Voltage-dependent calcium channel gamma-8 subunit GN=CACNG8 | 0.422 | 1.5E-02 |
| P11117 | Lysosomal acid phosphatase GN=ACP2 | 0.420 | 1.2E-02 |
| Q16288 | NT-3 growth factor receptor GN=NTRK3 | 0.420 | 2.8E-03 |
| P28222 | 5-hydroxytryptamine receptor 1B GN=HTR1B | 0.415 | 2.2E-05 |
| O14786 | Neuropilin-1 GN=NRP1 | 0.412 | 1.5E-05 |
| Q9HCJ2 | Leucine-rich repeat-containing protein 4C GN=LRRC4C | 0.400 | 4.0E-03 |
| O43300 | Leucine-rich repeat transmembrane neuronal protein 2 GN=LRRTM2 | 0.393 | 5.6E-03 |
| P08913 | Alpha-2A adrenergic receptor GN=ADRA2A | 0.378 | 3.3E-03 |
| Q9Y6N7 | Roundabout homolog 1 GN=ROBO1 | 0.361 | 5.8E-05 |
| Q15878 | Voltage-dependent R-type calcium channel subunit alpha-1E GN=CACNA1E | 0.348 | 2.5E-03 |
| Q32ZL2 | Lipid phosphate phosphatase-related protein type 5 GN=LPPR5 | 0.335 | 7.0E-04 |
| Q6ZN44 | Netrin receptor UNC5A GN=UNC5A | 0.331 | 2.0E-03 |
| Q7Z4T9 | Protein MAATS1 GN=MAATS1 | 0.330 | 6.3E-03 |
| Q86TG7 | Retrotransposon-derived protein PEG10 GN=PEG10 | 0.328 | 1.2E-03 |
| O43295 | SLIT-ROBO Rho GTPase-activating protein 3 GN=SRGAP3 | 0.326 | 1.6E-02 |
| Q7Z3F1 | Integral membrane protein GPR155 GN=GPR155 | 0.320 | 3.1E-03 |
| Q9H2B2 | Synaptotagmin-4 GN=SYT4 | 0.320 | 3.3E-02 |
| Q7L1I2 | Synaptic vesicle glycoprotein 2B GN=SV2B | 0.318 | 1.2E-02 |
| O60268 | Uncharacterized protein KIAA0513 GN=KIAA0513 | 0.317 | 7.9E-03 |
| Q6U841 | Sodium-driven chloride bicarbonate exchanger GN=SLC4A10 | 0.317 | 3.8E-07 |
| Q9H0Q3 | FXYD domain-containing ion transport regulator 6 GN=FXYD6 | 0.314 | 2.0E-02 |
| P43146 | Netrin receptor DCC GN=DCC | 0.310 | 4.3E-04 |
| O94772 | Lymphocyte antigen 6H GN=LY6H | 0.310 | 1.4E-04 |
| O60939 | Sodium channel subunit beta-2 GN=SCN2B | 0.308 | 1.5E-03 |
| Q92797 | Symplekin GN=SYMPK | 0.301 | 3.0E-02 |
| Q5T848 | Probable G-protein coupled receptor 158 GN=GPR158 | 0.299 | 1.8E-04 |
| P08138 | Tumor necrosis factor receptor superfamily member 16 GN=NGFR | 0.298 | 3.2E-03 |
| Q7Z6B7 | SLIT-ROBO Rho GTPase-activating protein 1 GN=SRGAP1 | 0.295 | 1.4E-02 |
| Q9NWQ8 | Phosphoprotein associated with glycosphingolipid-enriched microdomains 1 GN=PAG1 | 0.294 | 8.1E-03 |
| O15118 | Niemann-Pick C1 protein GN=NPC1 | 0.293 | 2.7E-02 |
| Q8N7J2 | APC membrane recruitment protein 2 GN=AMER2 | 0.291 | 1.9E-03 |
| Q6UXK2 | Immunoglobulin superfamily containing leucine-rich repeat protein 2 GN=ISLR2 | 0.289 | 3.1E-04 |
| Q96GW7 | Brevican core protein GN=BCAN | 0.287 | 7.9E-03 |
| P26006 | Integrin alpha-3 GN=ITGA3 | 0.287 | 8.2E-04 |
| O43424 | Glutamate receptor ionotropic, delta-2 GN=GRID2 | 0.287 | 5.3E-04 |
| A2A2Y4 | FERM domain-containing protein 3 GN=FRMD3 | 0.283 | 4.7E-02 |
| Q9NZV1 | Cysteine-rich motor neuron 1 protein GN=CRIM1 | 0.282 | 5.9E-04 |
| P16389 | Potassium voltage-gated channel subfamily A member 2 GN=KCNA2 | 0.281 | 1.6E-02 |
| O60486 | Plexin-C1 GN=PLXNC1 | 0.276 | 2.1E-02 |
| Q99784 | Noelin GN=OLFM1 | 0.276 | 7.6E-03 |
| Q6IAA8 | Ragulator complex protein LAMTOR1 GN=LAMTOR1 | 0.271 | 3.8E-03 |
| P34903 | Gamma-aminobutyric acid receptor subunit alpha-3 GN=GABRA3 | 0.270 | 1.7E-02 |
| Q9UNL2 | Translocon-associated protein subunit gamma GN=SSR3 | -0.273 | 4.3E-02 |
| P46782 | 40S ribosomal protein S5 GN=RPS5 | -0.276 | 1.5E-02 |
| Q5VU97 | VWFA and cache domain-containing protein 1 GN=CACHD1 | -0.277 | 1.3E-03 |
| Q9Y5G3 | Protocadherin gamma-B1 GN=PCDHGB1 | -0.285 | 2.2E-04 |
| Q9Y5H3 | Protocadherin gamma-A10 GN=PCDHGA10 | -0.286 | 2.0E-05 |
| Q53HI1 | Protein unc-50 homolog GN=UNC50 | -0.287 | 3.8E-02 |
| Q96FZ5 | CKLF-like MARVEL tm domain-containing protein 7 GN=CMTM7 | -0.288 | 1.3E-02 |
| Q9ULU8 | Calcium-dependent secretion activator 1 GN=CADPS | -0.290 | 2.1E-02 |
| Q96RF0 | Sorting nexin-18 GN=SNX18 | -0.291 | 2.2E-02 |
| Q8IZU9 | Kin of IRRE-like protein 3 GN=KIRREL3 | -0.291 | 1.6E-04 |
| Q8WY07 | Cationic amino acid transporter 3 GN=SLC7A3 | -0.298 | 1.8E-02 |
| Q9UN71 | Protocadherin gamma-B4 GN=PCDHGB4 | -0.299 | 3.0E-04 |
| P19623 | Spermidine synthase GN=SRM | -0.299 | 4.0E-03 |
| Q8IXJ6 | NAD-dependent protein deacetylase sirtuin-2 GN=SIRT2 | -0.306 | 6.3E-03 |
| P55285 | Cadherin-6 GN=CDH6 | -0.307 | 5.5E-04 |
| Q9Y4C0 | Neurexin-3 GN=NRXN3 | -0.307 | 1.6E-03 |
| P54851 | Epithelial membrane protein 2 GN=EMP2 | -0.308 | 1.7E-02 |
| Q8NG11 | Tetraspanin-14 GN=TSPAN14 | -0.311 | 9.4E-03 |
| O43657 | Tetraspanin-6 GN=TSPAN6 | -0.311 | 8.1E-04 |
| P06756 | Integrin alpha-V GN=ITGAV | -0.317 | 1.4E-04 |
| Q14DG7 | Transmembrane protein 132B GN=TMEM132B | -0.325 | 1.5E-02 |
| P78504 | Protein jagged-1 GN=JAG1 | -0.326 | 6.3E-04 |
| P47972 | Neuronal pentraxin-2 GN=NPTX2 | -0.327 | 1.2E-03 |
| O14763 | TNF receptor superfamily member 10B GN=TNFRSF10B | -0.328 | 2.5E-03 |
| P0DKB5 | Trophoblast glycoprotein-like GN=TPBGL PE=4 | -0.329 | 2.5E-02 |
| Q9UBG0 | C-type mannose receptor 2 GN=MRC2 | -0.330 | 8.7E-03 |
| Q9UI15 | Transgelin-3 GN=TAGLN3 | -0.331 | 1.5E-02 |
| P04921 | Glycophorin-C GN=GYPC | -0.331 | 1.4E-04 |
| Q92870 | Amyloid beta A4 precursor protein-binding FbM2 GN=APBB2 | -0.332 | 7.4E-03 |
| Q9NZW5 | MAGUK p55 subfamily member 6 GN=MPP6 | -0.332 | 2.0E-04 |
| P51153 | Ras-related protein Rab-13 GN=RAB13 | -0.337 | 3.6E-02 |
| P42574 | Caspase-3 GN=CASP3 | -0.337 | 2.9E-04 |
| P60033 | CD81 antigen GN=CD81 | -0.348 | 8.8E-04 |
| Q75V66 | Anoctamin-5 GN=ANO5 | -0.352 | 3.8E-03 |
| P39019 | 40S ribosomal protein S19 GN=RPS19 | -0.356 | 4.8E-02 |
| Q15392 | Delta(24)-sterol reductase GN=DHCR24 | -0.357 | 2.6E-02 |
| Q13797 | Integrin alpha-9 GN=ITGA9 | -0.359 | 1.4E-07 |
| Q9ULK6 | RING finger protein 150 GN=RNF150 | -0.359 | 5.3E-03 |
| Q2VWP7 | Protogenin GN=PRTG | -0.363 | 9.1E-03 |
| Q9Y4D7 | Plexin-D1 GN=PLXND1 | -0.365 | 2.1E-04 |
| Q9H813 | Transmembrane protein 206 GN=TMEM206 | -0.367 | 1.0E-06 |
| Q9UN70 | Protocadherin gamma-C3 GN=PCDHGC3 | -0.368 | 3.1E-03 |
| Q14831 | Metabotropic glutamate receptor 7 GN=GRM7 | -0.368 | 6.7E-04 |
| O95628 | CCR4-NOT transcription complex subunit 4 GN=CNOT4 | -0.370 | 1.3E-02 |
| Q14517 | Protocadherin Fat 1 GN=FAT1 | -0.371 | 4.2E-05 |
| O00401 | Neural Wiskott-Aldrich syndrome protein GN=WASL | -0.374 | 3.9E-02 |
| P13611 | Versican core protein GN=VCAN | -0.379 | 3.4E-02 |
| P29317 | Ephrin type-A receptor 2 GN=EPHA2 | -0.380 | 1.7E-03 |
| O14522 | Receptor-type tyrosine-protein phosphatase T GN=PTPRT | -0.380 | 1.2E-03 |
| Q08431 | Lactadherin GN=MFGE8 | -0.381 | 5.1E-03 |
| P08582 | Melanotransferrin GN=MFI2 | -0.381 | 9.0E-04 |
| Q01973 | Tyrosine-protein kinase transmembrane receptor ROR1 GN=ROR1 | -0.383 | 2.9E-06 |
| A6NHL2 | Tubulin alpha chain-like 3 GN=TUBAL3 | -0.390 | 2.1E-02 |
| P24821 | Tenascin GN=TNC | -0.397 | 2.6E-02 |
| Q13464 | Rho-associated protein kinase 1 GN=ROCK1 | -0.413 | 2.2E-02 |
| Q13237 | cGMP-dependent protein kinase 2 GN=PRKG2 | -0.414 | 4.7E-02 |
| Q9Y625 | Glypican-6 GN=GPC6 | -0.419 | 1.4E-05 |
| P00533 | Epidermal growth factor receptor GN=EGFR | -0.444 | 7.0E-05 |
| Q12841 | Follistatin-related protein 1 GN=FSTL1 | -0.447 | 3.1E-03 |
| Q16698 | 2,4-dienoyl-CoA reductase, mitochondrial GN=DECR1 | -0.454 | 7.0E-04 |
| P29320 | Ephrin type-A receptor 3 GN=EPHA3 | -0.463 | 7.6E-04 |
| A6NEH6 | Transmembrane protein 247 GN=TMEM247 PE=4 | -0.471 | 1.8E-03 |
| Q494V2 | Coiled-coil domain-containing protein 37 GN=CCDC37 | -0.479 | 3.4E-02 |
| P58335 | Anthrax toxin receptor 2 GN=ANTXR2 | -0.481 | 2.1E-05 |
| P08648 | Integrin alpha-5 GN=ITGA5 | -0.499 | 5.4E-06 |
| P23471 | Receptor-type tyrosine-protein phosphatase zeta GN=PTPRZ1 | -0.538 | 1.5E-07 |
| P35221 | Catenin alpha-1 GN=CTNNA1 | -0.541 | 1.7E-06 |
| P61073 | C-X-C chemokine receptor type 4 GN=CXCR4 | -0.552 | 5.1E-04 |
| Q6UXZ4 | Netrin receptor UNC5D GN=UNC5D | -0.583 | 8.9E-04 |
| P56373 | P2X purinoceptor 3 GN=P2RX3 | -0.584 | 1.1E-05 |
| Q9UKU6 | Thyrotropin-releasing hormone-degrading ectoenzyme GN=TRHDE | -0.587 | 5.7E-05 |
| Q92563 | Testican-2 GN=SPOCK2 | -0.592 | 1.4E-02 |
| Q99653 | Calcineurin B homologous protein 1 GN=CHP1 | -0.595 | 1.6E-02 |
| Q9NZU0 | Leucine-rich repeat transmembrane protein FLRT3 GN=FLRT3 | -0.598 | 9.2E-03 |
| Q02297 | Pro-neuregulin-1, membrane-bound isoform GN=NRG1 | -0.619 | 1.2E-05 |
| O14495 | Lipid phosphate phosphohydrolase 3 GN=PPAP2B | -0.732 | 7.9E-06 |
| Q99996 | A-kinase anchor protein 9 GN=AKAP9 | -0.793 | 3.4E-05 |
| Q96A83 | Collagen alpha-1(XXVI) chain GN=COL26A1 | -0.620 | 1.3E-02 |
| O75487 | Glypican-4 GN=GPC4 | -0.915 | 2.5E-04 |
