# Supplementary material for: Plasma membrane remodeling in GM2 gangliosidoses drives synaptic dysfunction
Source: PLoS Biol. 2025 Jul 3;23(7):e3003265. doi: 10.1371/journal.pbio.3003265 (PMC12251256; doi:10.1371/journal.pbio.3003265)
Supplement: S4 Table — Data for individual and combined comparisons are shown with those that satisfy the criteria of significant (<0.05) abundance change of >1.2 fold change (FC) are colored blue. (DOCX) [file pbio.3003265.s010.docx]

**S4 Table.** Targets identified in WCP-MS of ΔHEXA and ΔGLB1 compared with SCRM control cells at 28 dpi. Data for individual and combined comparisons are shown with those that satisfy the criteria of significant (<0.05) abundance change of >1.2 fold change (FC) are coloured blue.

|  |  | ΔHEXA vs SCRM | | ΔGLB1 vs SCRM | | Combined vs SCRM | |
| --- | --- | --- | --- | --- | --- | --- | --- |
| Gene ID | Description | Log_2_ FC | P value | Log_2_ FC | P value | Log_2_ FC | P value |
| Q9C0H2 | Protein tweety homolog 3 GN=TTYH3 | 1.629 | 4.5E-04 | 1.499 | 3.9E-04 | 1.564 | 3.3E-06 |
| O60637 | Tetraspanin-3 GN=TSPAN3 | 1.563 | 3.8E-03 | 1.367 | 7.5E-03 | 1.465 | 1.9E-04 |
| P07602 | Prosaposin GN=PSAP | 1.463 | 1.3E-03 | 1.281 | 1.8E-03 | 1.372 | 2.2E-05 |
| Q5HYI7 | Metaxin-3 GN=MTX3 | 1.204 | 2.4E-03 | 0.862 | 5.0E-03 | 1.033 | 9.9E-04 |
| P61916 | NPC intracellular cholesterol transporter 2 GN=NPC2 | 1.052 | 4.1E-03 | 1.029 | 1.7E-03 | 1.041 | 4.8E-05 |
| P08962 | CD63 antigen GN=CD63 | 1.048 | 8.2E-03 | 0.669 | 1.2E-01 | 0.859 | 1.7E-02 |
| Q96MP8 | BTB/POZ domain-containing protein KCTD7 GN=KCTD7 | 1.031 | 6.5E-04 | 0.727 | 3.7E-03 | 0.879 | 7.1E-04 |
| Q9H6Y7 | E3 ubiquitin-protein ligase RNF167 GN=RNF167 | 0.904 | 2.8E-02 | 0.804 | 8.2E-02 | 0.854 | 1.2E-02 |
| P61225 | Ras-related protein Rap-2b GN=RAP2B | 0.881 | 3.1E-02 | 0.809 | 4.7E-02 | 0.845 | 7.4E-03 |
| P38571 | Lysosomal acid lipase/cholesteryl ester hydrolase GN=LIPA | 0.825 | 6.0E-04 | 0.662 | 5.0E-02 | 0.744 | 2.6E-03 |
| Q99758 | Phospholipid-transporting ATPase ABCA3 GN=ABCA3 | 0.811 | 9.7E-03 | 0.549 | 6.0E-03 | 0.680 | 3.1E-03 |
| P41732 | Tetraspanin-7 GN=TSPAN7 | 0.799 | 2.9E-02 | 0.647 | 2.0E-02 | 0.723 | 3.3E-03 |
| O95415 | Membrane protein BRI3 GN=BRI3 | 0.786 | 1.7E-02 | 0.609 | 4.4E-02 | 0.698 | 3.9E-03 |
| P50897 | Palmitoyl-protein thioesterase 1 GN=PPT1 | 0.777 | 5.2E-03 | 0.733 | 4.9E-03 | 0.755 | 1.3E-04 |
| Q14108 | Lysosome membrane protein 2 GN=SCARB2 | 0.742 | 9.6E-03 | 0.508 | 2.0E-01 | 0.625 | 3.5E-02 |
| P53801 | Pituitary tumor-transforming gene 1 protein-interacting protein GN=PTTG1IP | 0.732 | 8.3E-03 | 0.742 | 2.2E-02 | 0.737 | 1.0E-03 |
| Q9NUN5 | Lysosomal cobalamin transport escort protein LMBD1 GN=LMBRD1 | 0.695 | 1.4E-02 | 0.514 | 3.4E-02 | 0.604 | 3.2E-03 |
| Q6UW56 | All-trans retinoic acid-induced differentiation factor GN=ATRAID | 0.655 | 5.1E-02 | 0.600 | 2.3E-02 | 0.627 | 3.8E-03 |
| Q86UL3 | Glycerol-3-phosphate acyltransferase 4 GN=GPAT4 | 0.648 | 1.0E-02 | 0.207 | 2.0E-01 | 0.428 | 7.4E-02 |
| Q8N4L2 | Type 2 phosphatidylinositol 4,5-bisphosphate 4-phosphatase GN=PIP4P2 | 0.635 | 2.7E-02 | 0.442 | 1.1E-01 | 0.539 | 1.1E-02 |
| Q8WZ42 | Titin GN=TTN | 0.629 | 4.6E-02 | 0.368 | 3.9E-01 | 0.498 | 1.2E-01 |
| P11279 | Lysosome-associated membrane glycoprotein 1 GN=LAMP1 | 0.591 | 3.9E-03 | 0.712 | 3.2E-03 | 0.651 | 2.4E-04 |
| Q969S3 | Cytoplasmic 60S subunit biogenesis factor ZNF622 GN=ZNF622 | 0.544 | 3.6E-02 | 0.763 | 5.9E-01 | 0.654 | 4.5E-01 |
| O95772 | STARD3 N-terminal-like protein GN=STARD3NL | 0.540 | 6.4E-02 | 0.313 | 1.2E-02 | 0.427 | 3.3E-02 |
| Q9NS87 | Kinesin-like protein KIF15 GN=KIF15 | 0.503 | 2.6E-02 | 0.595 | 4.4E-02 | 0.549 | 4.5E-03 |
| Q14849 | StAR-related lipid transfer protein 3 GN=STARD3 | 0.500 | 1.9E-02 | 0.292 | 5.9E-02 | 0.396 | 1.2E-02 |
| Q8WWI5 | Choline transporter-like protein 1 GN=SLC44A1 | 0.499 | 3.0E-02 | 0.453 | 1.1E-01 | 0.476 | 1.6E-02 |
| Q9BV68 | E3 ubiquitin-protein ligase RNF126 GN=RNF126 | 0.499 | 1.1E-02 | 0.362 | 2.4E-01 | 0.430 | 4.5E-02 |
| Q99590 | Protein SCAF11 GN=SCAF11 | 0.497 | 2.0E-02 | 0.414 | 2.6E-01 | 0.456 | 6.6E-02 |
| O43567 | E3 ubiquitin-protein ligase RNF13 GN=RNF13 | 0.491 | 1.0E-01 | 0.741 | 3.8E-02 | 0.616 | 1.5E-02 |
| Q9UGL1 | Lysine-specific demethylase 5B GN=KDM5B | 0.489 | 4.2E-03 | -0.003 | 9.9E-01 | 0.243 | 3.3E-01 |
| Q9Y664 | KICSTOR complex protein kaptin GN=KPTN | 0.460 | 6.3E-02 | 0.587 | 1.7E-02 | 0.524 | 6.0E-03 |
| Q8IY95 | Transmembrane protein 192 GN=TMEM192 | 0.460 | 2.1E-02 | 0.465 | 5.4E-03 | 0.462 | 4.8E-04 |
| Q14533 | Keratin, type II cuticular Hb1 GN=KRT81 | 0.455 | 1.8E-01 | 0.470 | 5.2E-03 | 0.462 | 3.0E-02 |
| Q9NVA4 | Transmembrane protein 184C GN=TMEM184C | 0.447 | 5.6E-03 | 0.469 | 7.4E-03 | 0.458 | 8.4E-05 |
| P07339 | Cathepsin D GN=CTSD | 0.445 | 2.2E-02 | 0.349 | 2.4E-02 | 0.397 | 2.1E-03 |
| Q86WC4 | Osteopetrosis-associated transmembrane protein 1 GN=OSTM1 | 0.435 | 7.6E-02 | 0.430 | 2.1E-02 | 0.432 | 9.3E-03 |
| Q9C026 | E3 ubiquitin-protein ligase TRIM9 GN=TRIM9 | 0.411 | 8.5E-03 | 0.066 | 7.4E-01 | 0.239 | 2.5E-01 |
| Q9UHA4 | Ragulator complex protein LAMTOR3 GN=LAMTOR3 | 0.411 | 1.4E-02 | 0.337 | 2.2E-02 | 0.374 | 1.9E-03 |
| P51948 | CDK-activating kinase assembly factor MAT1 GN=MNAT1 | 0.408 | 2.7E-02 | 0.445 | 2.6E-01 | 0.426 | 9.1E-02 |
| Q8N0U8 | Vitamin K epoxide reductase complex subunit 1-like protein 1 GN=VKORC1L1 | 0.406 | 9.4E-03 | 0.674 | 2.3E-01 | 0.540 | 1.3E-01 |
| Q96G21 | U3 small nucleolar ribonucleoprotein protein IMP4 GN=IMP4 | 0.401 | 2.0E-01 | 0.626 | 3.5E-02 | 0.513 | 4.1E-02 |
| Q9BVL2 | Nucleoporin p58/p45 GN=NUP58 | 0.399 | 1.1E-02 | 0.183 | 3.3E-01 | 0.291 | 7.3E-02 |
| Q86XN8 | RNA-binding protein MEX3D GN=MEX3D | 0.394 | 9.2E-03 | 0.417 | 1.5E-01 | 0.405 | 2.8E-02 |
| Q8TEW0 | Partitioning defective 3 homolog GN=PARD3 | 0.382 | 2.4E-02 | 0.275 | 1.4E-01 | 0.329 | 2.4E-02 |
| Q9NQ34 | Transmembrane protein 9B GN=TMEM9B | 0.380 | 2.5E-02 | 0.269 | 1.3E-01 | 0.324 | 2.0E-02 |
| Q68CP9 | AT-rich interactive domain-containing protein 2 GN=ARID2 | 0.379 | 1.7E-02 | 0.501 | 1.8E-01 | 0.440 | 6.1E-02 |
| Q9P2R6 | Arginine-glutamic acid dipeptide repeats protein GN=RERE | 0.378 | 4.0E-02 | 0.237 | 4.8E-01 | 0.307 | 1.8E-01 |
| P0DKX4 | Small integral membrane protein 18 GN=SMIM18 | 0.375 | 4.8E-02 | 0.319 | 2.4E-01 | 0.347 | 5.6E-02 |
| P10619 | Lysosomal protective protein GN=CTSA | 0.361 | 7.4E-02 | -0.667 | 1.7E-02 | -0.153 | 7.4E-01 |
| Q8TAB3 | Protocadherin-19 GN=PCDH19 | 0.358 | 4.7E-02 | 0.236 | 7.4E-02 | 0.297 | 2.4E-02 |
| Q5TCZ1 | SH3 and PX domain-containing protein 2A GN=SH3PXD2A | 0.357 | 6.7E-02 | 0.457 | 4.5E-02 | 0.407 | 5.6E-03 |
| Q9H7E9 | UPF0488 protein C8orf33 GN=C8orf33 | 0.356 | 2.6E-02 | 0.234 | 1.0E-01 | 0.295 | 1.6E-02 |
| Q15545 | Transcription initiation factor TFIID subunit 7 GN=TAF7 | 0.355 | 4.5E-02 | 0.233 | 2.9E-01 | 0.294 | 8.4E-02 |
| Q9UJV8 | Purine-rich element-binding protein gamma GN=PURG | 0.351 | 7.4E-03 | 0.276 | 8.0E-02 | 0.314 | 6.6E-03 |
| O43504 | Ragulator complex protein LAMTOR5 GN=LAMTOR5 | 0.350 | 2.3E-03 | 0.223 | 4.8E-01 | 0.287 | 1.7E-01 |
| Q8TBF4 | Zinc finger CCHC-type and RNA-binding motif-containing protein 1 GN=ZCRB1 | 0.349 | 1.9E-02 | 0.388 | 4.1E-02 | 0.368 | 5.2E-03 |
| Q8TB05 | UBA-like domain-containing protein 1 GN=UBALD1 | 0.340 | 5.1E-03 | 0.206 | 3.9E-01 | 0.273 | 1.0E-01 |
| Q92576 | PHD finger protein 3 GN=PHF3 | 0.333 | 2.9E-02 | 0.179 | 7.2E-02 | 0.256 | 2.3E-02 |
| Q9UJY4 | ADP-ribosylation factor-binding protein GGA2 GN=GGA2 | 0.328 | 1.9E-02 | 0.443 | 1.2E-02 | 0.386 | 2.1E-03 |
| Q9UPV9 | Trafficking kinesin-binding protein 1 GN=TRAK1 | 0.326 | 1.3E-02 | 0.231 | 2.3E-01 | 0.279 | 4.5E-02 |
| Q53FA7 | Quinone oxidoreductase PIG3 GN=TP53I3 | 0.324 | 1.5E-02 | 0.383 | 2.8E-01 | 0.354 | 1.1E-01 |
| Q8IU60 | m7GpppN-mRNA hydrolase GN=DCP2 | 0.324 | 2.0E-02 | 0.328 | 6.7E-02 | 0.326 | 8.7E-03 |
| P17661 | Desmin GN=DES | 0.322 | 1.5E-02 | 0.373 | 2.3E-01 | 0.347 | 7.9E-02 |
| Q96BM9 | ADP-ribosylation factor-like protein 8A GN=ARL8A | 0.321 | 1.7E-02 | 0.324 | 1.3E-02 | 0.323 | 2.7E-04 |
| Q14527 | Helicase-like transcription factor GN=HLTF | 0.319 | 2.9E-02 | 0.136 | 3.9E-01 | 0.228 | 1.2E-01 |
| Q8NFH8 | RalBP1-associated Eps domain-containing protein 2 GN=REPS2 | 0.316 | 2.4E-02 | 0.100 | 4.7E-01 | 0.208 | 1.4E-01 |
| P51798 | H(+)/Cl(-) exchange transporter 7 GN=CLCN7 | 0.316 | 4.3E-02 | 0.239 | 8.9E-02 | 0.278 | 1.8E-02 |
| Q9NP77 | RNA polymerase II subunit A C-terminal domain phosphatase SSU72 GN=SSU72 | 0.311 | 2.9E-02 | 0.125 | 3.4E-01 | 0.218 | 9.5E-02 |
| Q66LE6 | Serine/threonine-protein phosphatase 2A 55 kDa regulatory subunit B delta isoform GN=PPP2R2D | 0.309 | 1.6E-02 | 0.203 | 2.2E-01 | 0.256 | 4.3E-02 |
| P78537 | Biogenesis of lysosome-related organelles complex 1 subunit 1 GN=BLOC1S1 | 0.308 | 5.1E-04 | 0.254 | 2.5E-01 | 0.281 | 5.2E-02 |
| Q15052 | Rho guanine nucleotide exchange factor 6 GN=ARHGEF6 | 0.307 | 1.3E-02 | 0.602 | 9.3E-02 | 0.454 | 6.4E-02 |
| Q7Z5L9 | Interferon regulatory factor 2-binding protein 2 GN=IRF2BP2 | 0.306 | 2.2E-02 | 0.162 | 9.9E-02 | 0.234 | 3.3E-02 |
| Q92542 | Nicastrin GN=NCSTN | 0.305 | 6.7E-03 | 0.331 | 4.0E-03 | 0.318 | 4.9E-05 |
| P15586 | N-acetylglucosamine-6-sulfatase GN=GNS | 0.300 | 3.5E-02 | 0.398 | 2.1E-03 | 0.349 | 2.3E-03 |
| Q9NP58 | ATP-binding cassette sub-family B member 6 GN=ABCB6 | 0.297 | 4.6E-02 | 0.281 | 1.3E-01 | 0.289 | 2.6E-02 |
| Q9BRR6 | ADP-dependent glucokinase GN=ADPGK | 0.287 | 2.2E-02 | 0.032 | 9.0E-01 | 0.159 | 4.1E-01 |
| Q9NZ53 | Podocalyxin-like protein 2 GN=PODXL2 | 0.286 | 1.4E-01 | 0.322 | 2.8E-02 | 0.304 | 1.7E-02 |
| Q6P3X3 | Tetratricopeptide repeat protein 27 GN=TTC27 | 0.286 | 1.9E-02 | 0.341 | 5.2E-02 | 0.313 | 5.8E-03 |
| P40145 | Adenylate cyclase type 8 GN=ADCY8 | 0.284 | 4.3E-02 | 0.196 | 4.2E-01 | 0.240 | 1.5E-01 |
| P06132 | Uroporphyrinogen decarboxylase GN=UROD | 0.282 | 3.7E-02 | 0.180 | 1.3E-01 | 0.231 | 2.7E-02 |
| Q8N511 | Transmembrane protein 199 GN=TMEM199 | 0.281 | 3.6E-02 | 0.201 | 1.3E-01 | 0.241 | 2.8E-02 |
| Q92785 | Zinc finger protein ubi-d4 GN=DPF2 | 0.281 | 1.6E-03 | 0.374 | 1.0E-03 | 0.327 | 2.9E-04 |
| Q9NZE8 | Large ribosomal subunit protein bL35m GN=MRPL35 | 0.281 | 1.9E-02 | 0.211 | 2.9E-01 | 0.246 | 6.9E-02 |
| Q5T3F8 | CSC1-like protein 2 GN=TMEM63B | 0.280 | 1.7E-02 | 0.285 | 3.7E-02 | 0.282 | 2.2E-03 |
| P01111 | GTPase NRas GN=NRAS | 0.279 | 6.0E-03 | 0.168 | 1.5E-02 | 0.224 | 6.9E-03 |
| Q6IAA8 | Ragulator complex protein LAMTOR1 GN=LAMTOR1 | 0.278 | 3.9E-02 | 0.217 | 2.5E-01 | 0.248 | 6.1E-02 |
| Q9H8Y5 | tRNA endonuclease ANKZF1 GN=ANKZF1 | 0.276 | 3.3E-02 | 0.244 | 4.4E-01 | 0.260 | 2.0E-01 |
| P62847 | Small ribosomal subunit protein eS24 GN=RPS24 | 0.276 | 1.7E-02 | 0.137 | 3.2E-01 | 0.207 | 7.8E-02 |
| Q9NV79 | Protein-L-isoaspartate O-methyltransferase domain-containing protein 2 GN=PCMTD2 | 0.275 | 1.1E-02 | 0.136 | 8.1E-02 | 0.205 | 3.0E-02 |
| Q8WU68 | Splicing factor U2AF 26 kDa subunit GN=U2AF1L4 | 0.257 | 1.9E-01 | 0.291 | 2.5E-02 | 0.274 | 3.3E-02 |
| Q7Z2E3 | Aprataxin GN=APTX | 0.252 | 1.0E-01 | 0.270 | 4.3E-02 | 0.261 | 1.8E-02 |
| O60237 | Protein phosphatase 1 regulatory subunit 12B GN=PPP1R12B | 0.236 | 1.9E-01 | 0.442 | 3.0E-02 | 0.339 | 3.7E-02 |
| Q08722 | Leukocyte surface antigen CD47 GN=CD47 | 0.232 | 1.2E-01 | 0.529 | 3.2E-02 | 0.380 | 5.6E-02 |
| P16278 | Beta-galactosidase GN=GLB1 | 0.227 | 2.2E-01 | -2.025 | 1.4E-03 | -0.899 | 3.7E-01 |
| Q9BPX7 | UPF0415 protein C7orf25 GN=C7orf25 | 0.226 | 3.5E-01 | 0.449 | 8.6E-03 | 0.338 | 8.4E-02 |
| Q5VW32 | BRO1 domain-containing protein BROX GN=BROX | 0.210 | 1.9E-01 | 0.282 | 3.9E-02 | 0.246 | 4.5E-02 |
| P36896 | Activin receptor type-1B GN=ACVR1B | 0.190 | 3.8E-01 | 0.382 | 3.5E-02 | 0.286 | 1.2E-01 |
| Q5XKP0 | MICOS complex subunit MIC13 GN=MICOS13 | 0.182 | 1.1E-01 | 0.278 | 4.9E-02 | 0.230 | 1.8E-02 |
| Q8WXH2 | Junctophilin-3 GN=JPH3 | 0.178 | 6.1E-01 | 0.313 | 4.7E-02 | 0.245 | 2.9E-01 |
| P07858 | Cathepsin B GN=CTSB | 0.136 | 1.9E-01 | 0.284 | 4.3E-02 | 0.210 | 5.5E-02 |
| Q8NB90 | ATPase family gene 2 protein homolog A GN=AFG2A | 0.131 | 2.1E-01 | 0.307 | 4.1E-02 | 0.219 | 8.2E-02 |
| Q04771 | Activin receptor type-1 GN=ACVR1 | 0.129 | 5.7E-01 | 0.736 | 1.3E-02 | 0.433 | 1.8E-01 |
| Q9UGQ2 | Calcium channel flower homolog GN=CACFD1 | 0.125 | 1.9E-01 | 0.300 | 4.5E-02 | 0.212 | 8.2E-02 |
| Q9Y2W3 | Proton-associated sugar transporter A GN=SLC45A1 | 0.120 | 2.5E-01 | 0.287 | 3.7E-02 | 0.203 | 6.6E-02 |
| Q92520 | Protein FAM3C GN=FAM3C | 0.112 | 4.2E-01 | -0.969 | 1.0E-03 | -0.429 | 3.8E-01 |
| Q9BV23 | Monoacylglycerol lipase ABHD6 GN=ABHD6 | 0.065 | 3.2E-01 | 0.316 | 1.1E-02 | 0.190 | 1.4E-01 |
| P60903 | Protein S100-A10 GN=S100A10 | 0.060 | 7.8E-01 | 0.428 | 3.7E-02 | 0.244 | 2.8E-01 |
| Q92871 | Phosphomannomutase 1 GN=PMM1 | 0.016 | 8.4E-01 | -0.486 | 3.1E-02 | -0.235 | 3.3E-01 |
| Q9Y233 | cAMP and cAMP-inhibited cGMP 3',5'-cyclic phosphodiesterase 10A GN=PDE10A | -0.057 | 3.0E-01 | -0.395 | 3.7E-02 | -0.226 | 2.0E-01 |
| Q86SQ7 | Serologically defined colon cancer antigen 8 GN=SDCCAG8 | -0.066 | 8.4E-01 | -0.293 | 3.4E-02 | -0.180 | 4.3E-01 |
| Q5T7P8 | Synaptotagmin-6 GN=SYT6 | -0.083 | 2.4E-01 | -0.389 | 3.3E-03 | -0.236 | 1.3E-01 |
| Q68D20 | Protein PMS2CL GN=PMS2CL | -0.096 | 4.6E-01 | 0.300 | 1.1E-02 | 0.102 | 5.9E-01 |
| Q53H96 | Pyrroline-5-carboxylate reductase 3 GN=PYCR3 | -0.143 | 1.9E-01 | -0.321 | 4.9E-02 | -0.232 | 6.5E-02 |
| Q6PFW1 | Inositol hexakisphosphate and diphosphoinositol-pentakisphosphate kinase 1 GN=PPIP5K1 | -0.157 | 3.0E-01 | -0.599 | 3.9E-02 | -0.378 | 1.4E-01 |
| P41229 | Lysine-specific demethylase 5C GN=KDM5C | -0.247 | 1.8E-03 | -0.297 | 4.7E-03 | -0.272 | 1.7E-04 |
| Q5VVW2 | GTPase-activating Rap/Ran-GAP domain-like protein 3 GN=GARNL3 | -0.254 | 2.3E-01 | -0.444 | 4.3E-02 | -0.349 | 5.0E-02 |
| Q08828 | Adenylate cyclase type 1 GN=ADCY1 | -0.280 | 2.2E-01 | -0.413 | 2.6E-02 | -0.347 | 4.6E-02 |
| Q9Y2U9 | Kelch domain-containing protein 2 GN=KLHDC2 | -0.281 | 4.9E-03 | -0.197 | 5.9E-02 | -0.239 | 6.7E-03 |
| Q9Y4R8 | Telomere length regulation protein TEL2 homolog GN=TELO2 | -0.286 | 3.2E-02 | -0.263 | 2.2E-01 | -0.274 | 5.2E-02 |
| Q9BZM4 | UL16-binding protein 3 GN=ULBP3 | -0.291 | 9.1E-02 | -0.315 | 1.2E-02 | -0.303 | 1.1E-02 |
| Q9H857 | 5'-nucleotidase domain-containing protein 2 GN=NT5DC2 | -0.292 | 1.8E-02 | -0.206 | 4.2E-01 | -0.249 | 1.5E-01 |
| P15289 | Arylsulfatase A GN=ARSA | -0.294 | 3.5E-02 | -0.029 | 8.3E-01 | -0.162 | 2.8E-01 |
| Q6ZUT1 | Uncharacterized protein NKAPD1 GN=NKAPD1 | -0.307 | 2.5E-02 | 0.035 | 9.2E-01 | -0.136 | 6.1E-01 |
| Q658Y4 | Protein FAM91A1 GN=FAM91A1 | -0.382 | 7.6E-03 | -0.264 | 2.4E-01 | -0.323 | 4.6E-02 |
| Q9NPA2 | Matrix metalloproteinase-25 GN=MMP25 | -0.392 | 5.9E-02 | -0.432 | 4.9E-02 | -0.412 | 1.4E-02 |
| Q9Y6N8 | Cadherin-10 GN=CDH10 | -0.396 | 1.7E-02 | -0.471 | 2.3E-02 | -0.434 | 2.9E-03 |
| P43246 | DNA mismatch repair protein Msh2 GN=MSH2 | -0.397 | 2.5E-02 | -0.360 | 3.4E-01 | -0.378 | 1.2E-01 |
| Q86VY4 | Testis-specific Y-encoded-like protein 5 GN=TSPYL5 | -0.402 | 9.0E-02 | -0.307 | 3.6E-02 | -0.354 | 2.7E-02 |
| P49327 | Fatty acid synthase GN=FASN | -0.408 | 3.7E-02 | -0.516 | 2.3E-01 | -0.462 | 9.3E-02 |
| Q6ZSA7 | Leucine-rich repeat-containing protein 55 GN=LRRC55 | -0.418 | 2.6E-02 | -0.282 | 2.6E-01 | -0.350 | 5.6E-02 |
| Q3B7J2 | Glucose-fructose oxidoreductase domain-containing protein 2 GN=GFOD2 | -0.425 | 4.5E-02 | -0.155 | 3.6E-01 | -0.290 | 1.1E-01 |
| Q9P2Y4 | Zinc finger protein 219 GN=ZNF219 | -0.431 | 6.9E-03 | -0.070 | 3.7E-01 | -0.250 | 1.6E-01 |
| Q86YT6 | E3 ubiquitin-protein ligase MIB1 GN=MIB1 | -0.442 | 1.5E-02 | -0.153 | 3.7E-01 | -0.298 | 1.1E-01 |
| Q8IUH3 | RNA-binding protein 45 GN=RBM45 | -0.447 | 3.0E-02 | -0.311 | 3.5E-02 | -0.379 | 1.0E-02 |
| Q9NZM4 | BRD4-interacting chromatin-remodeling complex-associated protein GN=BICRA | -0.465 | 1.7E-02 | -0.428 | 1.1E-02 | -0.447 | 7.5E-04 |
| Q9P1Q0 | Vacuolar protein sorting-associated protein 54 GN=VPS54 | -0.471 | 2.0E-02 | 0.035 | 9.6E-01 | -0.218 | 6.3E-01 |
| Q9Y463 | Dual specificity tyrosine-phosphorylation-regulated kinase 1B GN=DYRK1B | -0.498 | 1.8E-03 | -0.004 | 9.8E-01 | -0.251 | 3.1E-01 |
| O76083 | High affinity cGMP-specific 3',5'-cyclic phosphodiesterase 9A GN=PDE9A | -0.518 | 4.4E-02 | -0.451 | 6.1E-02 | -0.484 | 6.9E-03 |
| Q63HM9 | PI-PLC X domain-containing protein 3 GN=PLCXD3 | -0.549 | 3.4E-02 | -0.384 | 3.1E-01 | -0.466 | 8.3E-02 |
| Q9P1Z2 | Calcium-binding and coiled-coil domain-containing protein 1 GN=CALCOCO1 | -0.631 | 3.3E-02 | -0.367 | 3.2E-01 | -0.499 | 8.8E-02 |
| Q5TC82 | Roquin-1 GN=RC3H1 | -0.642 | 1.5E-02 | -0.277 | 5.9E-02 | -0.460 | 4.0E-02 |
| P06865 | Beta-hexosaminidase subunit alpha GN=HEXA | -0.709 | 2.5E-03 | 0.361 | 1.8E-01 | -0.174 | 7.2E-01 |
