# Supplementary material for: Plasma membrane remodeling in GM2 gangliosidoses drives synaptic dysfunction
Source: PLoS Biol. 2025 Jul 3;23(7):e3003265. doi: 10.1371/journal.pbio.3003265 (PMC12251256; doi:10.1371/journal.pbio.3003265)
Supplement: S5 Table — (DOCX) [file pbio.3003265.s011.docx]

**S5 Table.** High confidence targets identified in PMP-MS of ΔHEXA and ΔGLB1 compared with SCRM control cells at 28 dpi.

| **Gene ID** | **Description** | **Significance p-value** | **Log_2_ Fold change** |
| --- | --- | --- | --- |
| Q14108 | Lysosome membrane protein 2 GN=SCARB2 | 2.1E-03 | 1.134298 |
| O60637 | Tetraspanin-3 GN=TSPAN3 | 2.0E-03 | 1.110642 |
| Q9C0H2 | Protein tweety homolog 3 GN=TTYH3 | 6.5E-04 | 0.927288 |
| Q96QD8 | Sodium-coupled neutral amino acid symporter 2 GN=SLC38A2 | 1.0E-04 | 0.584853 |
| P30044 | Peroxiredoxin-5, mitochondrial GN=PRDX5 | 2.9E-02 | 0.560899 |
| Q8NHG7 | Small VCP/p97-interacting protein GN=SVIP | 2.1E-02 | 0.509084 |
| Q8IV08 | 5'-3' exonuclease PLD3 GN=PLD3 | 4.9E-02 | 0.504786 |
| P51149 | Ras-related protein Rab-7a GN=RAB7A | 1.2E-02 | 0.481074 |
| Q9BVK6 | Transmembrane emp24 domain-containing protein 9 GN=TMED9 | 2.1E-02 | 0.440148 |
| Q99536 | Synaptic vesicle membrane protein VAT-1 homolog GN=VAT1 | 3.8E-02 | 0.422677 |
| P02656 | Apolipoprotein C-III GN=APOC3 | 5.0E-02 | 0.374355 |
| Q9H3Z4 | DnaJ homolog subfamily C member 5 GN=DNAJC5 | 1.8E-02 | 0.370676 |
| P45880 | Voltage-dependent anion-selective channel protein 2 GN=VDAC2 | 6.7E-03 | 0.349981 |
| P30048 | Thioredoxin-dependent peroxide reductase, mitochondrial GN=PRDX3 | 4.7E-02 | 0.345214 |
| Q9Y2Q0 | Phospholipid-transporting ATPase IA GN=ATP8A1 | 3.5E-02 | 0.278338 |
| P53794 | Sodium/myo-inositol cotransporter GN=SLC5A3 | 2.6E-02 | 0.2732 |
| Q93050 | V-type proton ATPase 116 kDa subunit a 1 GN=ATP6V0A1 | 1.4E-02 | -0.30675 |
| Q9NS85 | Carbonic anhydrase-related protein 10 GN=CA10 | 5.8E-04 | -0.37379 |
| O95180 | Voltage-dependent T-type calcium channel subunit alpha-1H GN=CACNA1H | 2.7E-02 | -0.43465 |
| Q9H2B2 | Synaptotagmin-4 GN=SYT4 | 1.9E-02 | -0.44747 |
