# Supplementary material for: Plasma membrane remodeling in GM2 gangliosidoses drives synaptic dysfunction
Source: PLoS Biol. 2025 Jul 3;23(7):e3003265. doi: 10.1371/journal.pbio.3003265 (PMC12251256; doi:10.1371/journal.pbio.3003265)
Supplement: S6 Table — (DOCX) [file pbio.3003265.s012.docx]

**S6 Table.** TaqMan Gene Expression Assay Probe Sets

| Gene | TaqMan Probe Set ID |
| --- | --- |
| GAPDH | Hs03929097_g1 |
| HEXA | Hs00166843_m1 |
| HEXB | Hs00166864_m1 |
| GLB1 | Hs01035163_m1 |
| NANOG | Hs02387400_g1 |
| OCT4 | Hs04260367_gH |
| SYP | Hs00300531_m1 |
| MAP2 | Hs00258900_m1 |
| β3-tubulin | Hs00801390_s1 |
| LAMP-1 | Hs00174766_m1 |
| PSAP | HS01551096_m1 |
| CD63 | Hs01041238_g1 |
| BSN | Hs01109512_m1 |
| GPC4 | Hs00155059_m1 |
| NRG1 | Hs01101538_m1 |
| CNTN5 | Hs00544269_m1 |
| CNTNAP4 | Hs00369159_m1 |
| Syt1 | Hs00194572_m1 |
