# Supplementary material for: Plasma membrane remodeling in GM2 gangliosidoses drives synaptic dysfunction
Source: PLoS Biol. 2025 Jul 3;23(7):e3003265. doi: 10.1371/journal.pbio.3003265 (PMC12251256; doi:10.1371/journal.pbio.3003265)
Supplement: S7 Table — (DOCX) [file pbio.3003265.s013.docx]

**S7 Table.** Purified lipids used as standards for PM glycan profiling

| Lipid | Product code | Supplier |
| --- | --- | --- |
| Phosphatidylcholine | 840051C-25mg | Avanti Polar Lipids |
| Cholesterol | C8667-500MG | Sigma-Aldrich |
| Rhodamine-Phosphatidyl ethanolamine | 810150C-1MG | Avanti Polar Lipids |
| GM3 | 860058P-5MG | Avanti Polar Lipids |
| GM2 | G8397-1MG | Sigma-Aldrich |
| GM1a | 860065P-1MG | Avanti Polar Lipids |
| GD1a | 860055P-1MG | Avanti Polar Lipids |
| GD1b | 860056P-1MG | Avanti Polar Lipids |
| GT1b | 860059P-1MG | Avanti Polar Lipids |
| GQ1b | 860086P-1MG | Avanti Polar Lipids |
