# Supplementary material for: Plasma membrane remodeling in GM2 gangliosidoses drives synaptic dysfunction
Source: PLoS Biol. 2025 Jul 3;23(7):e3003265. doi: 10.1371/journal.pbio.3003265 (PMC12251256; doi:10.1371/journal.pbio.3003265)
Supplement: Attachment — Submitted filename: 151284_1_rebuttal_3325368_st233r.pdf [file pbio.3003265.s015.pdf]

Full Revision

Manuscript number: RC-2024-02724
Corresponding author(s): Janet, Deane

1. General Statements [optional]

The goal of this study was to identify how dysregulation of lipid metabolism and the consequential dysfunction of the lysosome has a broader impact on neurons via remodelling of the plasma membrane with functional impact on synaptic signalling.

We thank the reviewers for their comments and have included substantial new data to strengthen the work by specifically addressing questions regarding the molecular mechanisms driving the proteomic and phenotypic changes observed in these disease models. We have generated a new ganglioside disease model (GM1 gangliosidosis) and demonstrated that the lysosomal exocytosis mechanism identified for GM2 gangliosidosis is a conserved mechanism that alters the PM proteome (**see new Figure 5**).

We have also carried out substantial additional experimental work to address the question of whether specific protein-lipid interactions drive some of these changes. We have preliminary data supporting this (included below) but we are not confident that these data are robust enough for inclusion in this manuscript. This work required substantial in vitro experiments including the expression and purification of several proteins for use in liposome binding assays. Although these data are promising, they have been challenging to reproduce and we would prefer to develop this work further for inclusion in a subsequent paper.

Although not requested by any reviewers we have also included substantial additional multielectrode array (MEA) data in Figure 4 to further support the phenotypic changes to electrical signalling seen in the Tay Sachs disease model.

We would like to note that even without these new data the reviewers highlighted that the "high-quality data presented significantly advance the field" and that the work "exposes key conceptual novelties" using "new insight" and "new tools" that shed "light on the complex pathophysiology that links lipid accumulation to neuronal dysfunction". And that this highlights "an underappreciated dimension of these diseases" allowing them to be "understood better thanks to this study". More generally the reviewers state that the work is of interest to both "clinicians and basic researchers" and is relevant to "broader fields in cellular and neurodegenerative biology".

Review COMMONS

2. Point-by-point description of the revisions

This section is mandatory. Please insert a point-by-point reply describing the revisions that were already carried out and included in the transferred manuscript.

Reviewer 1

• Confirmation of Neuronal Differentiation: To confirm neuronal differentiation in their i3N cell model, the authors show qPCR results indicating the expression of mature neuronal markers and the downregulation of stem cell markers by day 14. However, single-cell RNA sequencing (scRNA-seq) could provide a more detailed evaluation of the differentiation process, addressing the fine-grained cell-type composition within the cell population. Depending on the results, the authors might more precisely interpret functional data and assess the possible influence of increased GM2 levels on cell fate decisions.

The accumulation of GM2 may not be identical across all neurons and so it is possible that, although the neuronal populations as a whole display mature differentiation, individual cells may respond differently to the amount of lipid debris. However, there are several technical reasons why obtaining samples for scRNAseq is extremely challenging. By 14 dpi the separation of individual neurons from each other is very difficult as they are in a densely grown and highly attached and interconnected network. Furthermore, the individual neurons have a highly polarized differentiated morphology with long delicate axonal and dendritic projections, that are readily cleaved and lysed in the process of harvesting and dissociation to obtain single cell suspensions for FACS sorting. In neurons, mRNAs are also abundantly localised along the length of their neuritic projections [1], thus these damaged preparations would provide unreliably meaningful data. Alternatively, sufficiently isolated individual neurons show poor survival and do not mature. If these technical difficulties could be overcome, in order to monitor altered differentiation, it would be necessary to determine which timepoint was most relevant to capture differences between day 0 stem cells and day 28 when they are synchronously firing glutamatergic neuron cultures. For this analysis to be robust it would require sample preparation and analysis of multiple stages of the differentiation process. For all the reasons above we cannot address this reviewer's request.

• Mechanistic Links Between Lipid Accumulation and Proteomic Changes: The authors report specific proteome changes upon HEXA/B KO. What are the mechanistic links between lipid accumulation and proteomic changes? Is the overall degradative performance of lysosomes compromised? The authors note that certain proteins, such as TSPANs, can bind directly to GSL headgroups. Clarifying whether the observed proteomic changes result from specific, direct lipid-protein interactions versus indirect effects could strengthen the argument for targeted lipid-mediated proteomic shifts.

In response to these questions, we have carried out substantial additional experimental work testing the lipid interactions of some of the proteins that are most altered in their abundance at

the PM. We focussed on the top non-lysosomal proteins as we are proposing that the lysosomal ones are primarily changed due to lysosomal exocytosis, suggesting the non-lysosomal are the best candidates for direct GSL-binding. To robustly identify specific lipid-protein interactions is highly challenging but something we have demonstrated previously [2].

In vitro lipid-binding assays require expression and purification of the proteins of interest to then be used in liposome pulldown experiments using liposomes of defined composition. As we are most interested in the specificity of the headgroup interaction we focussed on producing the extracellular portions of these proteins that would be predicted to bind these headgroups (again this is a strategy we have successfully used previously [2]). We expressed and purified the extracellular domains of three top non-lysosomal hits: CNTNAP4, CNTN5 and NTRK2 (**Fig. R1A**). These purified proteins were used in liposome-binding assays using liposomes composed of different sphingolipids and gangliosides (**Fig. R1B**). These data demonstrate that the GPI-anchored protein CNTN5 and its potential binding partner CNTNAP4 bind promiscuously to different headgroups. This may be consistent with their being incorporated into GSL-rich membrane microdomains via the GPI-anchor. Interestingly, in this assay NTRK2 demonstrates specific and substantial binding to GM2, with some weaker binding to GD3.

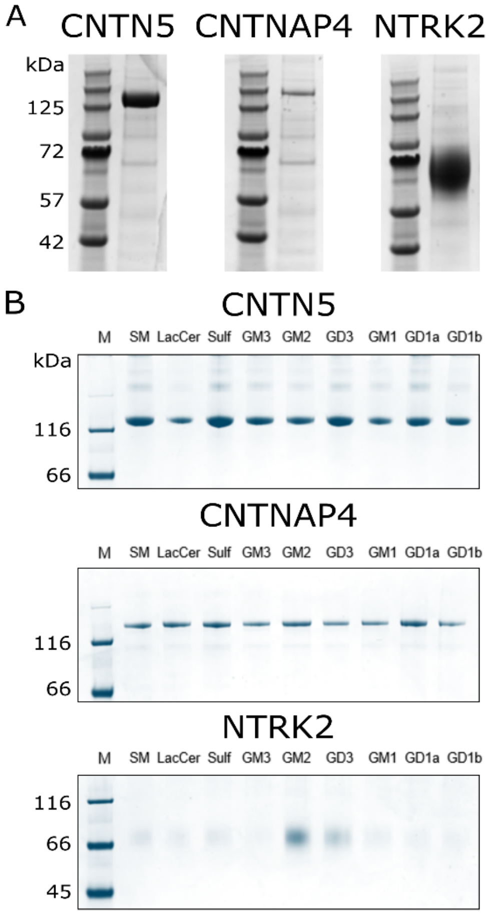

Figure R1. GSL-binding by top non-lysosomal PMP hits. (A) Coomassie-stained SDS-PAGE of CNTN5, CNTNAP4 and NTRK2 extracellular domains following expression in HEK293F cells and purification by affinity and size-exclusion chromatography. **(B)** Liposome pulldown assays using liposomes composed of 80% PC and 20% different sphingolipids including sphingomyelin (SM), lactosylceramide (LacCer), sulfatide (Sulf) and specific gangliosides including GM2.

These data support that the increased abundance of NTRK2 at the PM could be driven by direct interactions with the same lipid that is accumulating at the PM. As exciting and compelling as these data are, we have subsequently been unable to repeat this observation for NTRK2. We are unsure why and have tried several different strategies to test this interaction, but at this stage with only an N=1 for this observation we do not feel confident to include these data in the manuscript.

We intend to pursue this further using a range of alternative techniques and protein constructs but this will take substantial additional time and effort that we feel go beyond the scope of this current manuscript.

Additionally, does this phenomenon extend to other sphingolipidoses (e.g., Gaucher disease)? Comparing the proteomes of i3N cells across different sphingolipidoses could reveal whether the accumulation of distinct GSLs produces unique or shared proteomic profiles, highlighting similarities or specificities across lysosomal storage disorders.

We agree with the reviewer that this is an interesting and important question and had intended to do this as follow-up work in a future publication. However, in the interests of addressing this point here, we are including additional data we have generated from a new i3N model of GM1 gangliosidosis. As for the GM2 gangliosidosis models, we used CRISPRi to knockdown GLB1 and have confirmed this KD by q-PCR. We have also profiled the GSL composition and quantified the increased GM1 abundance. We have followed this up with both whole-cell and PM proteomics. We have presented comparative proteomics of the two models and demonstrated that they both result in significant accumulation of lysosomal proteins both in cells and at the PM. This shared proteomic profile is consistent with lysosomal exocytosis being a conserved mechanism driving altered PM composition in these diseases. We have included this work as an additional results section and an additional figure (**Figure 5**) as well as expanding the discussion. For this analysis we collected mass spec data at 28 dpi based on our observations in the paper that electrical signalling was synchronised at this point (Fig 4). In the text we discuss additional changes in these new WCP data such as the appearance of other trafficking molecules such as Arl8a that further support a lysosomal exocytosis mechanism.

In terms of the unique proteomic profiles of these diseases, the read depth of the PMP data in this case was not sufficient to confidently identify differences between the two gangliosidosis models and therefore we intend to pursue this work with additional LSDs in future studies to be included in a follow-up paper.

In terms of mechanistic links between lipid accumulation and proteome changes, we feel these new data provide substantial additional support that the appearance of lysosomal proteins at the PM is driven by lysosomal exocytosis and have preliminary data supporting that some non-lysosomal protein changes may be driven by altered protein-lipid interactions.

• Impact of Increased PM GM2 Levels on Endocytic Pathways: Along similar lines, the authors show differences in the PM proteome and in the representation of specific PM lipid domain-associated proteins. As some of these proteins are turned over by mechanisms involving lipid domain-dependent endocytosis, the authors might want to examine the effect of increased PM GM2 levels on various endocytic pathways.

We thank the reviewer for this suggestion and have attempted assays monitoring endocytosis using several approaches including the uptake of fluorescently labelled bovine serum albumin (DQ-BSA) [3–5]. These endocytosis assays are well established in standard cell lines such as HeLa cells. Despite several attempts by us to get this working in neurons using multiple alternative readouts (microscopy and plate-based fluorescence) we have been unable to measure changes in endocytosis. Exploration of alternative methods to probe Clathrin-independent/dynamin-

independent endocytosis (CLIC/GEEC) suggests these pathways are difficult to observe by fluorescence microscopy as there is minimal concentration of cargo proteins during the formation of carriers before endocytosis [6]. As an alternative strategy to probe changes in lipid-domain dependent endocytosis we have analysed the proteomics data for changes in galectins but no changes were identified in the data. We also explored available tools for modulating lysosomal exocytosis and monitoring lysosomal movement including activating TRPML1 to trigger exocytosis and activating ABCA3 to drive more lipid accumulation [7–10]. Similarly to the endocytosis assays above, these were not translatable to neurons in our hands due to a range of challenges including increased toxicity of these drugs on this cell type. We have made a substantial effort to try and address these questions and have conferred with colleagues who have also reported difficulties in establishing these assays in neurons. We are keen to continue to pursue this question but due to the technical challenges we feel this work lies beyond the scope of the current manuscript.

• Multifaceted Nature of Gangliosidoses as PM Disorders: The manuscript presents an important perspective by reframing gangliosidoses as multifaceted PM disorders that disrupt neuronal function and membrane composition. By further elaborating on the connection between membrane lipid alterations, neuronal excitability, and synaptic composition, and by exploring the interplay with lysosomal dysfunction, the authors could provide a richer understanding of gangliosidoses and GSL function in general.

We appreciate that the reviewer agrees with us that reframing gangliosidoses as more complex multifaceted diseases is important. We are not sure if there is a request here for more elaboration in the text but based on the new data included in the paper, we have expanded some of the discussion around these points. We are very enthusiastic to continue to probe the connections and interplay as described by the reviewer and this is the focus of our ongoing studies.

Reviewer 2

1. T-tests and one-way ANOVAs were used, but it is not clear if datasets were tested for normality and equal standard deviations. Please add these details. If data are not normal or standard deviations are unequal, other tests will have to be used.

All graphs were checked for normality and variance in standard deviation and for figure 1F, where the data was not normally distributed, a Kruskal-Wallace test was used in place of a one-way ANOVA. All significantly different results are now labelled on graphs and the relevant tests described in the figure legends. This has also all been updated in the Supplementary data.

2. It needs to be clearly explained how many data points were used for statistical analyses and what the data points were. E.g., N=3 independent experiments on 3 different days, each done in n=3 different wells, total n=9. Each well can be considered a biological replicate, but it's of lesser value than the "big Ns" done on different days. The authors can choose different ways of defining

their N/n numbers, but it has to be transparent. The bar graphs would ideally display the data points.

All figure legends now clearly explain N and n numbers used in experiments. Individual data points are displayed on qPCR graphs where N and n are mixed, with shapes denoting the biological repeat (N). In addition to clarification in figure legends, N and n numbers are described in the methods sections where appropriate.

For completeness we also include here details of these N/n numbers.

- For the q-PCR experiments, technical triplicates (repeats on the same day, n=3) were carried out for 3 separate biological replicates on different days (N=3). We have changed how these data are plotted to clarify this.
- For the activity assays, N=3 biological replicates were carried out on cell lysates from cultures grown on different days.
- For the microscopy analysis, coverslips from N=3 biological replicates on different days were used. n=2 coverslips per N were used to generate 15 images per N.
- For the glycan analysis, N=3 independent cell pellets were prepared on different days.
- For the proteomics experiments, these were done as N=3 independent cell cultures grown and prepared on different days. Specifically, one of each cell line SCRM, HEXA-1, HEXA-2, HEXB-1 and HEXB-2 were grown and harvested or biotinylated at a time (for WCP or PMP), with repeats on different days. These N=3 were then combined for the ΔHEX-A/B lines to provide N=12 biological repeats for disease cell lines to be compared to N=3 biological repeats for "SCRM" control cell lines.
- For calcium imaging, n=4 wells for each of SCRM, ΔHEXA-1 and ΔHEXB1 were averaged and the mean from each was used to provide n=3 data points across two biological repeats of this experiment, N=2.
- For the MEA data, we now include substantially more data than in the original manuscript (see comments at the top of this document). This is now N=3 biological replicates across n=52 wells over a time period from 38-45 dpi.
- The N/n values and statistical tests have also all been updated in the Supplementary data.

3. There should be a comment on how statistical power was calculated upfront and if not: how N/n numbers were chosen ("based on similar expts in the past").

N/n numbers, as detailed above, were chosen based on previous experiments by ourselves and others, as well as recommended practice [2,11–15]. Typically, these papers do not describe the statistical power upfront. We have added statements to this effect and relevant references to the methods section of the manuscript.

4. "This suggests that some of the proteins that are accumulating in these diseases are specifically products of lipid accumulation rather than a product of general lysosomal dysfunction.

In further support of this, several lysosomal proteins including V-type ATPases (ATP6 family), mannose-6-phosphate receptor (M6PR) and biogenesis of lysosomal organelle complex subunits (BLOC1) are quantified in the WCP but are not increased in abundance." This part is confusing. It seems like the authors observe an accumulation of endolysosomes in general (page 6), but then only certain endolysosomal proteins accumulate - and the authors speculate that this is due to decreased degradation or enhanced translation (mRNA levels are unaffected). This question should be addressed better, ideally experimentally: are endolysosomes accumulating in general or not? And what defines the endolysosomal proteins that accumulate vs. those that don't? How is that regulated?

Recently published work has identified that late endosomes/lysosomes do not possess one composition; they are dynamically remodelled and there is substantial heterogeneity in the composition of different lysosomes [16,17]. While some components, such as LAMP1 and Cathepsin D, are common across all lysosomal compartments there is considerable heterogeneity in the composition of these organelles. These studies also demonstrate that in disease-relevant conditions or upon drug treatment, lysosomes change their protein composition. For example, in a LIPL-4 KO mouse model they observe an increased abundance of Ragulator complex components, similarly to the increase in LAMTOR3 seen in our new 28 dpi WCP data for GM1 and GM2 gangliosidoses. Interestingly, in this study they demonstrate that lysosomal lipolysis leads to bigger changes in lysosomal protein composition than other pro-longevity mechanisms [17]. Another recent paper looking at a different lysosomal storage disease in microglia with accumulating GSLs and cholesterol has also identified abundance changes in a subset of lysosomal proteins including several we observe here including TTYH3, NPC1, PSAP and TSPAN7 [18]. Beyond proteomic analyses, the experimental tools for identifying these different populations are currently very limited, but these published studies support that it is possible to have accumulation of what we define as lysosomes by IF (using LAMP1 or lysotracker) but for the proteomic analysis to identify increased abundance of only a subset of lysosomal proteins.

These papers do not identify or speculate on how these differences are regulated. Analysis of the changes in our WCP as well as the new data for GM1 gangliosidoses support that the proteins that are most changed in response to GSL accumulation are membrane proteins involved in lipid and cholesterol binding and transport (New Fig 2D and 5E and see response below). This specific enrichment suggests that the changes are directly linked to the lipid changes, thus our suggestion that these accumulate due to a need for the cell to process these lipids but also that they may get "trapped" in the membrane whorls such that they are not efficiently degraded.

We have included the references above and a more detailed description of lysosomal heterogeneity into the main text to help address the reviewer's questions.

5. Fig. 1D: The GO terms are confusing. Why are there more proteins in the category lysosomal membrane than lysosome as a whole? Other categories seem to be overlapping as well.

Review COMMONS

We apologize for the confusion; this graph does not display protein counts it is the adjusted P values for the enrichment of the term. To make this clearer, the DAVID analysis graphs are now presented in a new format. We present in this new graph the false discovery rate (FDR) (adjusted P value) which is a measure of the significance of whether that GO term is specifically enriched in the dataset. We have also expanded the GO term analysis to include molecular function and biological process descriptors in addition to the cellular component originally described. For full clarity, to the right of each term we include the number of significant hits that have this term, that being the number of proteins that are contributing to this GO term enrichment.

6. Fig. 2C/3A: It'd be good to also show the hits that don't match the expectation/pathways of interest.

We provide a full list in the Supplementary Information of all hits that are considered significant allowing the reader to access this information without having to download the datasets from PRIDE. We did not label all hits in these panels to avoid cluttering the image. In the main text we have focused on those that clearly fall within related categories or pathways as we feel that several "hits" in the same area represents a more compelling and confident assessment of the data. Several of the additional hits not mentioned in the main text do still match the expectations/pathways. For example, one of the top hits not labelled in the WCP is GPR155 (a cholesterol binding protein at the lysosomal membrane) and one of the top unlabelled hits in the PMP data is OPCML (a GPI-anchored protein that clusters in GSL-rich microdomains). There are some, such as KITLG (up in the PMP data), that we don't currently have a hypothesis for why/how they change, but we are reluctant to describe and speculate upon additional isolated/orphan hits in the main text when these have not been further validated.

7. Fig. 3: It is not intuitive that synaptic proteins in particular would accumulate at the plasma membrane due to the lipid storage defect. Are they mis-trafficked or are they at synaptic membranes? That could, e.g, be addressed by isolating synaptosomes. And why this selectivity for synaptic proteins? Neurons should have more plasma membrane that is not synaptic. And, e.g, the release of lysosomal material should not happen at synapses (and lysosomes should not deliver synaptic proteins to the PM, unless there is a failure to degrade them).

We agree that synapses represent a relatively small proportion of the entire PM of neurons, but synapses are particularly enriched with glycosphingolipids where they affect synaptogenesis and synaptic transmission [19–22]. For these reasons we think that some synaptic proteins are particularly sensitive to these lipid changes as they are localised in GSL-rich membrane microdomains. We have now clarified this point in the text. We have also further clarified that we were not proposing that lysosomal proteins are present at the synapses. We observed that lysosomal proteins are enriched at the PM and this may be more generally across the whole PM, while the changes to synaptic proteins may or may not be localised at the synapse. We apologise for the confusion and have modified the text at the end of the PM proteomics results section to make this clearer.

To try and address experimentally the question of whether these proteins are at synapses, we have attempted synaptosome enrichment. However, lysosomal compartments co-sedimented with synaptosomes during the preparation – LAMP1 staining was enriched in the synaptosome preparations of all samples including SCRM controls. Therefore, we cannot distinguish these compartments which is particularly problematic in this disease model.

(7. Continued) Or is there an effect on synaptic vesicles? Are there more? Do they deliver their cargo more readily? Or is there a failure to do endocytosis of synaptic proteins, and that's why the accumulate? What is the connection between SVs and endolysosomes? More clarity would be good here.

We do think that there is an effect on synaptic vesicles particularly as the SV proteins SYT1 and SV2b are significantly increased in abundance at the PM suggesting they are not being internalized normally. Furthermore, the new WCP data going out to 28 dpi for both GM1 and GM2 gangliosidoses have identified a significant increase in Arl8a which plays a shared role in lysosomal and SV anterograde trafficking [23,24]. Whilst previously thought of as discrete pathways, evidence now suggests that endolysosomal and SV recycling pathways form a continuum with several shared proteins involved in the fusion, trafficking and sorting in both pathways [25]. Arl8a provides a good example of an adaptor protein that functions in both pathways and also when overexpressed results in enhanced neurotransmission consistent with our studies [26]. We have adjusted the discussion text to include a description of the links between SVs and endolysosomal trafficking and the potential shared role Arl8a may be playing in both pathways.

Regarding the question of whether there are more SVs or not, this is hard to determine directly as they are particularly small (~50 nm) and difficult to visualise or specifically stain for using microscopy. Not all SV-associated proteins are increased in the PMP data, for example SNAP25 and several other synaptotagmins are not changed in the 28 dpi data for both gangliosidosis models. We hope in the future to address SV changes more directly with higher resolution imaging such as electron microscopy or cryo-tomography but cannot currently confidently answer these specific questions.

8. Fig. 4: The assumption that there is more synaptic activity because there are more synaptic proteins at the membrane seems to be plausible, but also speculative at this point.

We have modified the text at the end of this results section to highlight that this is a speculative link.

9. The possible contribution of glial cells should at least be discussed.

We mention potential deleterious effects on bystander cells including other neurons, astrocytes and microglia in the second last paragraph of the discussion. In response to this request we have expanded and modified this text.

Minor: there are some typos etc.

Although no specific examples were listed, we have endeavored to find and correct typos, we have also checked for English spelling (not American) throughout.

Reviewer 3

1. Results section, 1st paragraph- to develop disease models- -- Please add cellular models as we already have KO mouse models.

This has been added to the text.

2. It was not clear what was the percentage of mutation success with their CRISPR technique.

The CRISPR method employed here was CRISPRi so there is no mutation of the genome. Instead, inactive/dead-Cas9 is targeted to the promotor/early exon of the HEXA or HEXB gene to inhibit mRNA production. We have included qPCR data to demonstrate the extent of the KD for two different guides to each of these genes in Fig 1.

3. Will the anti-GM2 antibody be available for other researchers? The researcher details needs to be clarified.

The anti-GM2 antibody is not commercial available and was generated by one of the co-authors. We invite scientists with an interest in this antibody to contact the corresponding author for details.

4. Hex activity assay was shown in 1C, but it was not clear that it is MUG or MUGS.

We apologise for this and have relabelled these activity assay graphs and expanded the legend text to clarify how these two substrates were used to distinguish the two different KD lines. We also corrected a small mistake in the methods section.

5. Is there a significance in Figure 2 B, 4A, 4B,4C and 4E?

Based on additional requests from reviewer 2 we have added significance indicators and details of significance tests for several panels in Figures 1-5 including 2B and 4B. For 4A we do not state a significant difference, we use these data to select a timepoint (28 dpi) where all cell lines have synchronous (correlated) signal. The data in Figure 4C and D have been substantially updated and expanded. Analysis of the data in 4C is plotted in 4D where we show significance. For 4E we are stating that the applied stimulation (white triangles) stimulates the HEXA cells every time but the SCRM do not respond to each stimulation. It is not clear how we would quantify this difference and there is no precedent for doing this in the MEA literature or by the Axion company who

provided the instrument. We have also included additional references for best practice when analysing MEA data.

REFERENCES

1. Mofatteh M. mRNA localization and local translation in neurons. AIMS Neurosci. 2020;7: 299–310. doi:10.3934/Neuroscience.2020016

2. McKie SJ, Nicholson AS, Smith E, Fawke S, Caroe ER, Williamson JC, et al. Altered plasma membrane abundance of the sulfatide-binding protein NF155 links glycosphingolipid imbalances to demyelination. Proc Natl Acad Sci U S A. 2023;120: e2218823120. doi:10.1073/pnas.2218823120

3. Marwaha R, Sharma M. DQ-Red BSA Trafficking Assay in Cultured Cells to Assess Cargo Delivery to Lysosomes. Bio Protoc. 2017;7: e2571. doi:10.21769/BioProtoc.2571

4. gustavo.parfitt. Lysosome proteolysis analysis with DQ-BSA. 2022 [cited 13 Feb 2025]. Available: https://www.protocols.io/view/lysosome-proteolysis-analysis-with-dq-bsa-cgjxtupn

5. Fernandez-Mosquera L, Yambire KF, Couto R, Pereyra L, Pabis K, Ponsford AH, et al. Mitochondrial respiratory chain deficiency inhibits lysosomal hydrolysis. Autophagy. 2019;15: 1572–1591. doi:10.1080/15548627.2019.1586256

6. Rennick JJ, Johnston APR, Parton RG. Key principles and methods for studying the endocytosis of biological and nanoparticle therapeutics. Nat Nanotechnol. 2021;16: 266–276. doi:10.1038/s41565-021-00858-8

7. Pastore N, Annunziata F, Colonna R, Maffia V, Giuliano T, Custode BM, et al. Increased expression or activation of TRPML1 reduces hepatic storage of toxic Z alpha-1 antitrypsin. Molecular Therapy. 2023;31: 2651–2661. doi:10.1016/j.ymthe.2023.06.018

8. Zhang H, Wang Y, Wang R, Zhang X, Chen H. TRPML1 agonist ML-SA5 mitigates uranium-induced nephrotoxicity via promoting lysosomal exocytosis. Biomedicine & Pharmacotherapy. 2024;181: 117728. doi:10.1016/j.biopha.2024.117728

9. Shen D, Wang X, Li X, Zhang X, Yao Z, Dibble S, et al. Lipid Storage Disorders Block Lysosomal Trafficking By Inhibiting TRP Channel and Calcium Release. Nat Commun. 2012;3: 731. doi:10.1038/ncomms1735

10. Wünkhaus D, Tang R, Nyame K, Laqtom NN, Schweizer M, Scotto Rosato A, et al. TRPML1 activation ameliorates lysosomal phenotypes in CLN3 deficient retinal pigment epithelial cells. Sci Rep. 2024;14: 17469. doi:10.1038/s41598-024-67479-8

11. Zlamalova E, Rodger C, Greco F, Cheers SR, Kleniuk J, Nadadhur AG, et al. Atlastin-1 regulates endosomal tubulation and lysosomal proteolysis in human cortical neurons. Neurobiol Dis. 2024;199: 106556. doi:10.1016/j.nbd.2024.106556

12. Anderson GSF, Ballester-Beltran J, Giotopoulos G, Guerrero JA, Surget S, Williamson JC, et al. Unbiased cell surface proteomics identifies SEMA4A as an effective immunotherapy target for myeloma. Blood. 2022;139: 2471–2482. doi:10.1182/blood.2021015161

13. Mossink B, Verboven AHA, Hugte EJH van, Gunnewiek TMK, Parodi G, Linda K, et al. Human neuronal networks on micro-electrode arrays are a highly robust tool to study disease-specific genotype-phenotype correlations in vitro. Stem Cell Reports. 2021;16: 2182–2196. doi:10.1016/j.stemcr.2021.07.001

14. McCready FP, Gordillo-Sampedro S, Pradeepan K, Martinez-Trujillo J, Ellis J. Multielectrode Arrays for Functional Phenotyping of Neurons from Induced Pluripotent Stem Cell Models of Neurodevelopmental Disorders. Biology. 2022;11: 316. doi:10.3390/biology11020316

15. Weaver S, Dube S, Mir A, Qin J, Sun G, Ramakrishnan R, et al. Taking qPCR to a higher level: Analysis of CNV reveals the power of high throughput qPCR to enhance quantitative resolution. Methods. 2010;50: 271–276. doi:10.1016/j.ymeth.2010.01.003

16. Bond C, Hugelier S, Xing J, Sorokina EM, Lakadamyali M. Heterogeneity of late endosome/lysosomes shown by multiplexed DNA-PAINT imaging. J Cell Biol. 2025;224: e202403116. doi:10.1083/jcb.202403116

17. Yu Y, Gao SM, Guan Y, Hu P-W, Zhang Q, Liu J, et al. Organelle proteomic profiling reveals lysosomal heterogeneity in association with longevity. Elife. 2024;13: e85214. doi:10.7554/eLife.85214

18. Yasa S, Butz ES, Colombo A, Chandrachud U, Montore L, Tschirner S, et al. Loss of CLN3 in microglia leads to impaired lipid metabolism and myelin turnover. Commun Biol. 2024;7: 1373. doi:10.1038/s42003-024-07057-w

19. Sipione S, Monyror J, Galleguillos D, Steinberg N, Kadam V. Gangliosides in the Brain: Physiology, Pathophysiology and Therapeutic Applications. Front Neurosci. 2020;14: 572965. doi:10.3389/fnins.2020.572965

20. Svennerholm L. Gangliosides and Synaptic Transmission. In: Svennerholm L, Mandel P, Dreyfus H, Urban P-F, editors. Structure and Function of Gangliosides. Boston, MA: Springer US; 1980. pp. 533–544. doi:10.1007/978-1-4684-7844-0_46

21. Palmano K, Rowan A, Guillermo R, Guan J, McJarrow P. The role of gangliosides in neurodevelopment. Nutrients. 2015;7: 3891–3913. doi:10.3390/nu7053891

22. Hering H, Lin C-C, Sheng M. Lipid rafts in the maintenance of synapses, dendritic spines, and surface AMPA receptor stability. J Neurosci. 2003;23: 3262–3271. doi:10.1523/JNEUROSCI.23-08-03262.2003

23. Rizalar FS, Lucht MT, Petzoldt A, Kong S, Sun J, Vines JH, et al. Phosphatidylinositol 3,5-bisphosphate facilitates axonal vesicle transport and presynapse assembly. Science. 2023;382: 223–230. doi:10.1126/science.adg1075

24. Klassen MP, Wu YE, Maeder CI, Nakae I, Cueva JG, Lehrman EK, et al. An Arf-like small G protein, ARL-8, promotes the axonal transport of presynaptic cargoes by suppressing vesicle aggregation. Neuron. 2010;66: 710–723. doi:10.1016/j.neuron.2010.04.033

25. Ivanova D, Cousin MA. Synaptic Vesicle Recycling and the Endolysosomal System: A Reappraisal of Form and Function. Front Synaptic Neurosci. 2022;14: 826098. doi:10.3389/fnsyn.2022.826098

26. Vukoja A, Rey U, Petzoldt AG, Ott C, Vollweiter D, Quentin C, et al. Presynaptic Biogenesis Requires Axonal Transport of Lysosome-Related Vesicles. Neuron. 2018;99: 1216-1232.e7. doi:10.1016/j.neuron.2018.08.004

27. Saheki Y, De Camilli P. Synaptic Vesicle Endocytosis. Cold Spring Harb Perspect Biol. 2012;4: a005645. doi:10.1101/cshperspect.a005645
